# Directing the Differentiation of Parthenogenetic Stem Cells into Tenocytes for Tissue‐Engineered Tendon Regeneration

**DOI:** 10.5966/sctm.2015-0334

**Published:** 2016-08-18

**Authors:** Wei Liu, Lu Yin, Xingrong Yan, Jihong Cui, Wenguang Liu, Yang Rao, Mei Sun, Qi Wei, Fulin Chen

**Affiliations:** ^1^ Rege Lab of Tissue Engineering, Faculty of Life Science, Northwest University, Xi'an, People's Republic of China; ^2^ Medical Experiment Center, Shaanxi University of Chinese Medicine, Xi'an‐Xianyang New Economic Zone, People's Republic of China; ^3^ Provincial Key Laboratory of Biotechnology of Shaanxi, Northwest University, Xi'an, People's Republic of China

**Keywords:** Parthenogenetic stem cells, Differentiation, Mechanical stretch, Tenocytes, Tendon regeneration

## Abstract

Uniparental parthenogenesis yields pluripotent stem cells without the political and ethical concerns surrounding the use of embryonic stem cells (ESCs) for biomedical applications. In the current study, we hypothesized that parthenogenetic stem cells (pSCs) could be directed to differentiate into tenocytes and applied for tissue‐engineered tendon. We showed that pSCs displayed fundamental properties similar to those of ESCs, including pluripotency, clonogenicity, and self‐renewal capacity. pSCs spontaneously differentiated into parthenogenetic mesenchymal stem cells (pMSCs), which were positive for mesenchymal stem cell surface markers and possessed osteogenic, chondrogenic, and adipogenic potential. Then, mechanical stretch was applied to improve the tenogenic differentiation of pMSCs, as indicated by the expression of tenogenic‐specific markers and an increasing *COL1A1*:*3A1* ratio. The pSC‐derived tenocytes could proliferate and secrete extracellular matrix on the surface of poly(lactic‐co‐glycolic) acid scaffolds. Finally, engineered tendon‐like tissue was successfully generated after in vivo heterotopic implantation of a tenocyte‐scaffold composite. In conclusion, our experiment introduced an effective and practical strategy for applying pSCs for tendon regeneration. Stem Cells Translational Medicine *2017;6:196–208*

**Significance Statement**

This study examined whether parthenogenetic stem cells exhibited properties similar to ESCs, including pluripotency, clonogenicity, self‐renewal, and in vitro and in vivo differentiation capacity. By sequential differentiation of parthenogenetic stem cells (pSCs), parthenogenetic mesenchymal stem cells had been established and further induced through cyclic mechanical stimulation. Cell proliferation, tendon‐specific marker, and extracellular matrix‐enhanced constructs were assessed in vitro and in vivo. Collectively, the study's data indicated that pSCs are an attractive cell source for tissue‐engineered tendon.

## Introduction

The critical function of the tendon is to transfer force from muscle to bone to support body movement. Tendon injury due to acute or chronic trauma is a common disorder and may cause pain and body morbidity in both professional and private life 1 2 3 4. Injured tendon has limited self‐healing capacity owing to its avascularity and acellularity. Currently, clinical options to treat tendon injuries include autograft and allograft transplantation. However, harvesting of autografts will create a secondary injury site, and transplantation of allografts carries the risk of immune rejection and infectious pathogen transmission 5 6 7. Furthermore, transplanted grafts rarely restore the structural and functional integrity of injured tendons owing to poor healing at the tendon‐tendon or tendon‐bone interface.

Cell therapy‐based approaches (e.g., tissue engineering) show great potential for tendon regeneration and repair. Considerable efforts have been made to employ various types of cells for tendon regeneration and repair. However, to date, no clinically practical approaches have been developed. Tenocytes may be an adequate cell source for tendon regeneration. However, despite donor site morbidity, limited numbers of cells can be obtained from explanted tissue because tendons are relatively acellular 1, and increasing passage number may result in phenotypic drift 8. Alternatively, dermal fibroblasts are more readily available through a simple dermal biopsy and are amenable to culture. Dermal fibroblasts could adhere to and proliferate on biomaterials and have been shown to synthesize many components of the extracellular matrix (ECM) 9, 10. The key concern with the use of fibroblast is scar formation, which may significantly influence the mechanical strength of the tendon 11.

Mesenchymal stem cells (MSCs) are characterized by their extensive proliferative ability and the potential to differentiate along various lineages, including bone, cartilage, adipose tissue, and tendon. Previous studies have shown that MSCs could form tendon‐like tissue and improve the mechanical properties of injured tendon 12. However, long‐term in vivo observations have demonstrated that ossification frequently occurs in the repaired tissue; this may impair the structure and function of the tendon 13, 14. Ouyang's group established a strategy to induce differentiation of embryonic stem cells (ESCs) for tendon regeneration 15. The results suggested that the progenitor cells derived from ESCs could promote tendon regeneration by secreting fetal tendon‐specific matrix and growth factors. Furthermore, no teratoma or ossification was observed in the newly formed tissue 15. However, harvesting of ESCs involves the destruction of viable embryos, limiting its use due to political and ethical concerns. Therefore, new cell‐based model systems for tissue engineering and regeneration are needed.

Parthenogenesis refers to the embryonic development of eggs activated without fertilization. Mammalian oocytes can be artificially stimulated to develop into diploid nonembryonic blastocysts, and parthenogenetic stem cells (pSCs) can be obtained from the blastocoel inner cell mass. In primates, parthenotes are unable to grow into viable fetuses because genetic defects affect proper placenta formation 16 17 18 19 20 21. pSCs are more histocompatible than other transplanted cells due to the presence of homozygous human leukocyte antigen (HLA) genotypes. These common HLA haplotype‐matched pSCs may reduce the risk of immune rejection after transplantation of their differentiated derivatives, thus offering significant advantages for application to cell‐based therapies compared with ESCs 22 23 24. pSCs can develop into retinal pigment epithelium‐like cells 25, muscle‐like and bone‐like cells 26, neuronal cells 27, 28, and hepatocytes 29. Moreover, cardiomyocytes derived from pSCs have been reported to facilitate engineering of myocardium and have been shown to enhance regional myocardial function after myocardial damage. *MHC*‐haploidentical pSC allografts have been shown to be immunologically accepted in related and unrelated recipients 30. However, no studies to date have identified the tenogenic differentiation capacity of pSCs.

Here, we examined whether pSCs exhibited properties similar to ESCs, including pluripotency, clonogenicity, self‐renewal, and in vitro and in vivo differentiation capacity. By sequential differentiation of pSCs, parthenogenetic mesenchymal stem cells (pMSCs) had been established and further induced through cyclic mechanical stimulation. Cell proliferation, tendon‐specific marker, and ECM‐enhanced constructs were assessed in vitro and in vivo. Collectively, our data indicated that pSCs are an attractive cell source for tissue‐engineered tendon.

## Materials and Methods

### Animals

Six‐week‐old nude mice and C57BL/6 mice were purchased from the Shanghai Experimental Animal Centre, Chinese Academy of Sciences (Shanghai, China, http://english.cas.cn). All animal studies and protocols were carried out following the guidelines of the Animal Holding Unit of Northwest University.

### Derivation and Expansion of pMSCs

The 5‐day embryoid bodies (EBs) were plated onto dishes coated with 0.1% gelatin (Sigma‐Aldrich, St. Louis, MO, https://www.sigmaaldrich.com) and cultured with complete growth medium for 7 days with medium changes every 3 days. Spindle‐shaped cells were observed in the outgrowths. The cells were then selectively separated by cell scrapers and collected the scrapes to sediment at the bottom of the tube, subcultured in MesenCult MSC Basal Medium (Stemcell, Cambridge, U.K., https://www.stemcell.com) for 21 days. The culture medium was changed every other day. Cells were subcultured for additional two to three passages before use.

### Flow Cytometry

pMSCs and embryonic MSCs (eMSCs; passage 3) were dissociated and washed with phosphate‐buffered saline (PBS). We immunolabeled 1 × 10^5^ cells with 1 mg of phycoerythrin (PE)‐ or fluorescein isothiocyanate (FITC)‐conjugated rat antimouse monoclonal antibodies against CD13, CD29, CD34, CD44, CD45, CD90.2, CD105, CD117, and CD133 (Becton Dickinson, BD Biosciences, San Jose, CA, http://www.bdbiosciences.com) for 1 hour at 37°C. For negative controls, isotype‐matched negative control antibodies (Becton Dickinson) were used under the same conditions. After three washes, the cells were examined using a FACSCalibur flow cytometer, and the data were analyzed with Cell Quest software (Beckton Dickinson).

### Induction Toward Multiple Cell Lineages and Special Staining

pMSCs and eMSCs were induced to differentiate into osteogenic, chondrogenic, and adipogenic lineages. For osteogenic differentiation, both types of cells were cultured in osteogenic medium (DMEM [Dulbecco's modified Eagle's medium] supplemented with 10% fetal bovine serum (FBS), 50 μM ascorbic acid‐2‐phosphate, 10 mM β‐glycerophosphate, and 50 nM dexamethasone [all from Sigma‐Aldrich]). The medium was changed every 3 days. After 3 weeks, the cells were fixed in 4% paraformaldehyde and processed for Alizarin Red S and Von Kossa staining. For chondrogenic differentiation, cells were cultured in chondrogenic medium (DMEM supplemented with 10% FBS, 50 μM ascorbic acid‐2‐phosphate [Sigma‐Aldrich], and 10 ng/ml transforming growth factor [TGF]‐β1 [R&D Systems, Minneapolis, MN, https://www.rndsystems.com]). Cells were processed for Safranin O and immunocytochemistry staining for *COL2A1* and Aggrecan after 3 weeks. For adipogenic differentiation, cells were exposed to adipogenic medium (DMEM supplemented with 10% FBS, 1 μM dexamethasone, 200 μM indomethacin [Sigma‐Aldrich], 10 μg/ml insulin [Sigma‐Aldrich], and 0.5 mM methylisobutylxanthine [Sigma‐Aldrich]) for 4 weeks. The medium was changed every 3 days. After 4 weeks, the cells were processed for Oil Red O and immunocytochemistry staining of aP2 and C/EBP.

### Gene Expression Analysis by QuantitativePolymerase Chain Reaction

Total RNA was extracted from the cells using an RNA Isolation Reagent (Takara Bio Inc., Kusatsu, Japan, http://www.takara-bio.com) according to the manufacturer's protocol. The extracted RNA was quantified using a GeneQuant pro (GE Healthcare Life Sciences, Chicago, IL, http://www.gehealthcare.com). A RevertAid First Strand cDNA Synthesis Kit (Thermo Fisher Scientific, Waltham, MA, https://www.thermofisher.com) was used to convert the RNA template into cDNA. Quantitative polymerase chain reaction (q‐PCR) was performed using a Bio‐Rad Q‐PCR system (Bio‐Rad, Hercules, CA, http://www.bio-rad.com). The relative levels of gene expression were conducted, using the comparative ΔΔCT method, with β‐actin as an internal control and normalized to the control group. The primers used are listed in the supplemental online data.

### Immunocytochemistry

pMSCs, induced chondrogenic, and adipogenic lineages were cultured on glass coverslips. Mechanical stimulation groups were seeded onto collagen type 1‐coated BioFlex plates. Both groups were fixed with 4% paraformaldehyde in PBS for 1 hour at 4°C then permeabilized for 8–15 minutes in PBS containing 0.5% Triton‐X 100, blocked for 2 hours in PBS containing 10% bovine serum albumin (BSA) and immunostained with primary antibodies overnight at 4°C. Primary antibodies used were as follows: antivimentin, anti‐α‐actin, anti‐*COL2A1* (collagen 2), anti‐*COL1A1* (collagen 1), anti‐*COL3A1* (collagen 3), anti‐*COL5A1* (collagen 5), anti‐tenomodulin (*TNMD*), anti‐tenascin‐C (*TNC*) (Santa Cruz Biotechnology, Santa Cruz, CA, http://www.scbt.com), anti‐Aggrecan, anti‐*HSP47*, anti‐bone morphogenetic protein 2 (*BMP2*), anti‐bone morphogenetic protein 13 (*BMP13*), anti‐*EYA2*, antimyogenin (*MYOG*), antimyosin heavy chain (*MHC*), and anti‐scleraxis (*SCX*; Abcam, Cambridge, United Kingdom, http://www.abcam.com). After 2 washes with PBS, the slides was incubated with suitable Alexa Fluor 488‐ or Alexa Fluor 594‐labeled secondary antibodies (Thermo Fisher) in the dark for 1 hour at 37°C. After 2 washes, nuclei were counterstained with 4′,6‐diamidino‐2‐phenylindole (DAPI; Thermo Fisher). Images were taken with a laser confocal microscope (FV1000; Olympus Corporation, Tokyo, Japan, http://www.olympus-global.com). For negative controls, isotype‐matched negative control antibodies (Santa Cruz Biotechnology) were used under the same conditions.

### Mechanical Stretch Stimulation

Mechanical stretch was applied to induce cellular tenogenic lineage differentiation with the Flexcell FX‐5000 Tension System (Flexcell International Corporation, Burlington, NC, http://www.flexcellint.com). pMSCs, dermal fibroblasts, and bone marrow stem cells (BMSCs) were seeded onto collagen type 1‐coated BioFlex plates at a density of 1 × 10^5^ cells per well. When the cultures reached approximately 70%–80% confluence, the cells were subjected to cyclic mechanical stretch with 10% elongation for 24 hours or 10 days (16 hours/day). Each cycle consisted of a 10‐second stretch and 10‐second relaxation. Control cultures were grown under the same conditions but without stretch.

### Western Blotting

pMSCs were subjected to cyclic mechanical stretch with 10% elongation for 24 hours or 10 days (16 hours/day). Total protein was quantified using a BCA Assay Kit (Thermo Fisher). Protein blotting was performed in a Mini‐PROTEAN Tetra (Bio‐Rad) at a constant current of 110 V for 60–120 minutes. Membranes were blocked with 5% (w/v) BSA (Merck, Darmstadt, Germany, http://www.merckgroup.com) for 2 hours. Membranes were then incubated overnight with primary antibodies, followed by application of appropriate horseradish peroxidase (HRP)‐conjugated secondary antibodies (Santa Cruz Biotechnology) for 1 hour. Proteins were detected using the ECL Advance chemiluminescent substrate (GE Healthcare Life Sciences). The primary antibodies used were as follows: anti‐*COL1A1*, anti‐*COL3A1*, anti‐*TNMD*, anti‐*SCX*, anti‐glyceraldehyde 3‐phosphate dehydrogenase (Santa Cruz Biotechnology), and anti‐*EYA2*.

### WST‐1 Cell Proliferation Assay

After the cells were subjected to cyclic mechanical stretch with 10% elongation for 10 days (16 hours/day), cell viability was analyzed using 4‐[3‐(4Iodophenyl)‐2‐(4‐nitrophenyl)‐2H‐5‐tetrazolio]‐1, 3‐benzene disulfonate (WST‐1) assays. For this procedure, cells were seeded in 96‐well plates at different cell densities from 1000 cells per 100 μl in DMEM supplemented with 10% FBS in quintuplicate for each cell density. A 10‐µl volume of cell proliferation reagent WST‐1 (Roche, Berlin, Germany, http://www.roche.com) was added to each well, and plates were incubated at 37°C using 5% CO_2_ in a humidified incubator for up to 10 days. The absorbance was measured every 24 hours in a microplate reader (BioTek Synergy HT, Winooski, VT, http://www.biotek.com) at 450 nm.

### CM‐Dil Cell Tracking and Detection

Cells were labeled with CM‐Dil (Thermo Fisher) according to the manufacturer's instructions before seeding into poly (lactic‐coglycolic) acid (PLGA) scaffolds to track cells in vitro and in vivo.

### In Vitro Evaluation of Cell Growth on Scaffolds

For in vitro evaluation of cell growth, 1‐cm crimp‐like PLGA (LA/GA 10:90; Ethicon, Somerville, NJ, http://www.ethicon.com) scaffolds were sterilized with 75% ethanol and air dried before use. Next, 1 × 10^5^ PDTs (pMSCs after 10 days of stretch) in 10 μl medium (DMEM supplemented with 10% FBS) were then seeded onto scaffolds in 6‐well plates. Two milliliters of medium was added 4 hours later to facilitate cell attachment. Eighteen cell‐scaffold constructs were prepared. Culture media were changed every 2–3 days during the 15‐day culture period. Three specimens were harvested at predesignated time intervals of 5, 10, and 15 days. DNA was isolated from each construct with DNAiso reagents (Takara Bio) according to the manufacturer's protocol. The extracted DNA was quantified using a GeneQuant pro (GE Healthcare Life Sciences).

### Scanning Electron Microscopy Examination

After observation by laser confocal microscope, three specimens from each scaffold group at different time intervals were prepared for scanning electron microscopy (SEM) examination. The specimens were prefixed with 2% glutaraldehyde, postfixed with 1% osmic acid, sputter‐coated with gold (Bal‐Tec, Philips, Eindhoven, The Netherlands, http://www.philips.com), and examined with a scanning electron microscope (Philips‐XL‐30; Philips) to observe cell attachment.

### In Vivo Transplantation and Observation

PDTs‐PLGA constructs were transplanted subcutaneously into the backs of 6‐week‐old nude mice (*n* = 8, each mouse implanted with 1 construct). The constructs were harvested at 6 and 12 weeks after implantation for histological (H&E staining and Masson's trichrome staining), immunohistochemical, and electron microscopic examination. For immunohistochemical analysis, the sections were immunolabeled with primary antibodies, including *COL1A1*, *COL3A1*, *COL5A1*, *TNC*, *TNMD*, *SCX*, *HSP47*, *EYA2*, *BMP2*, and BMP13. Isotype‐matched negative control antibodies were used under the same conditions as negative controls. Then the slides were incubated with suitable Alexa Fluor 488‐ or Alexa Fluor 594‐labeled secondary antibodies (Thermo Fisher). Nuclei were counterstained with DAPI (Thermo Fisher). Images were taken with a laser confocal microscope.

### Transmission Electron Microscopy Examination

The newly formed tendon tissues obtained from the in vivo experiments were fixed in fixative solution (2.5% glutaraldehyde and 2% formaldehyde) embedded in epoxy resin. The prepared samples were sectioned to 70–90‐nm thicknesses. Ultrathin sections were stained in 3% aqueous uranyl acetate and then in Sato triple lead stain before examination using an FEI CM12 Electron Microscope (FEI Company, Hillsboro, OR, https://www.fei.com). Image‐Pro Plus Analysis Software (version 6.0; Media Cybernetics, Rockville, MD, http://www.mediacy.com) was applied to quantify fibril diameter. Briefly, for each sample, three images and approximately 90–100 collagen fibrils from each image were randomly selected for measuring and obtaining the average fibril diameter of each examined sample.

### Statistical Analysis

The data are expressed as means ± SDs from three replicate experiments. Data sets that involved more than two groups were assessed by one‐way analysis of variance (ANOVA) followed by Newman‐Keuls post hoc tests (Fig. 2A; supplemental online Figs. 19, 20). In the figures, the data with different superscript letters are significantly different based on post hoc ANOVA statistical analysis. Differences with *p* < .05 were considered significant.

## Results

### Characterization of pSCs and Generation of MSCs From pSCs

The commonly used criteria to define stem cells are pluripotency, clonogenicity, and self‐renewal. We first tested morphology and alkaline phosphatase (ALP) activity ( supplemental online Fig. 1) and presence of stemness markers ( supplemental online Fig. 2), and we found no differences in PSCs with J1 cells. A small population (approximately 14%–20%) of pSCs formed adherent cell colony ( supplemental online Figs. 3, 4). WST‐1 assays showed that J1 cells proliferated more rapidly than pSCs at all densities, as assessed at six time points. The molecular basis of these patterns of proliferation demonstrated that pSCs maintained low expression of the *IGF2* gene (1.09 ± 0.21 vs. 3.26 ± 0.4, respectively; *p* < .001; supplemental online Fig. 5) may result in a decline in proliferative rates.

We compared pSCs, J1 lines for their EB formation and multilineage differentiation capability in EB cultures using (a) limiting dilution for EB formation efficiencies ( supplemental online Table 1); (b) TdT‐mediated dUTP nick‐end labeling (TUNEL) for EB apoptotic nuclei ( supplemental online Fig. 6); (c) Q‐PCR for ectodermal, mesodermal, endodermal, and epithelial‐mesenchymal transition (EMT) markers ( supplemental online Figs. 7, 9); (d) immunofluorescence staining for early mesodermal (stem cells antigen‐1 [SCA‐1] and CD34), mesodermal (*NT5E*, *MYOG*, α‐actin) and EMT (N‐cadherin, vimentin, and E‐cadherin markers [ supplemental online Fig. 8]); (e) the process coincided with a multiplex migration of spindle‐shaped, brick‐shaped cells and formed beating cardiomyocytes ( supplemental online Fig. 10; supplemental online Movie 1); and (f) teratoma formation after subcutaneous injection in nude mice ( supplemental online Fig. 11) .

**Figure 1:**
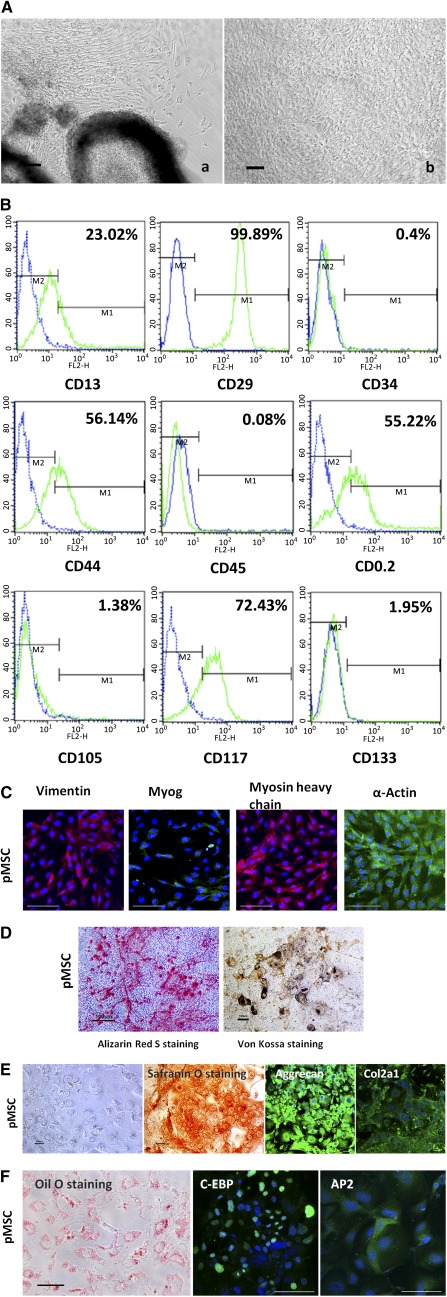
Generation of MSCs from pSCs. **(A):** Outgrowths from 5‐day EBs were collected and subcultured 7 days after plating **(Aa)**. Expansion of MSCs from plating scraped cells with MesenCult; most cells of passage 3–4 exhibited fibroblast‐like morphology **(Ab)**. **(B):** Representative histograms of cell surface marker expression on pMSCs (passage 3) analyzed by flow cytometry. Labeled cells are represented by the green line (M1), and relevant isotype‐matched cells are depicted by the blue line (M2). **(C):** Immunocytochemistry staining for mesodermal markers in pMSCs (passage 3). Antibody staining (green and red), with DAPI nuclear staining (blue). **(D):** Alizarin Red S and Von Kossa staining showed the osteogenic differentiation of pMSCs. **(E):** Chondrogenic differentiation of pMSCs. Chondrogenic differentiation was assessed by Safranin O staining. **(F):** Oil Red O staining and the expression of C/EBP and AP2 showing adipogenic differentiation of pMSCs. Scale bar = 100 μm. Abbreviations: DAPI, 4′,6‐diamidino‐2‐phenylindole; EB, embryoid body; MSC, mesenchymal stem cell; pMSC, parthenogenetic mesenchymal stem cell; pSC, parthenogenetic stem cell.

According to gene expression levels for mesodermal commitment, the outgrowth of spindle‐shaped cells was selectively isolated with a cell scraper 7 days after EB plating, and the scraped cells were subsequently expanded in MesenCult MSC Basal Medium to obtain MSCs (Fig. 1A; supplemental online Fig. 12). The population of cells obtained was passaged at 14–21 days after plating. After two to three additional passages, the cells exhibited a uniform spindle‐shaped pattern.

pMSCs and eMSCs had similar surface marker expression profiles. The pMSCs expressed CD29 (integrin β‐1), with the majority of cells expressing CD44 (hyaluronate receptor; 56.14%), CD90.2 (thymocyte differentiation antigen‐1β; 55.22%), and CD105 (endoglin, Src Homology 2 [SH2]; 72.47%). CD13 (alanyl aminopeptidase) was expressed in 23.02% of cells. These data indicated that the cells possessed a mesenchymal identity. pMSCs and eMSCs were negative for CD45 (leukocyte common antigen), CD34 (hematopoietic progenitor cell antigen), CD117 (c‐kit), and CD133 (aminopeptidase‐N), indicating that they were not of hematopoietic origin (Fig. 1B; supplemental online Fig. 13). Immunocytochemistry staining showed that the pMSCs consistently expressed *MYOG*, *MHC*, vimentin, and α‐actin (Fig. 1C; supplemental online Fig. 14).

The multidifferentiation potential of pMSCs toward osteogenic, chondrogenic, and adipogenic lineages was analyzed. Q‐PCR analysis showed that the expression of osteogenic markers, including ALP, bone sialoprotein (IBSP), osteocalcin (BGLAP1), secreted phosphoprotein 1 (OPN), and runt‐related transcription factor 2 (RUNX2), was increased. Strong staining for Alizarin Red S and Von Kossa 21 days after induction demonstrated accumulated mineralization, indicating that these cells had osteogenic potential after induction (Fig. 1D; supplemental online Fig. 15). Safranin O, immunocytochemistry staining, and Q‐PCR verified the chondrogenic lineage differentiation (Fig. 1E; supplemental online Fig. 16). The cells also had the capacity to undergo adipogenic differentiation. This was evident through the accumulation of lipid vacuoles and upregulated expression of the adipogenic markers aP2 and C/EBP after induction (Fig. 1F; supplemental online Fig. 17).

### Mechanical Stimulation Enhanced the Tenogenic Phenotype of pMSCs

**Figure 2:**
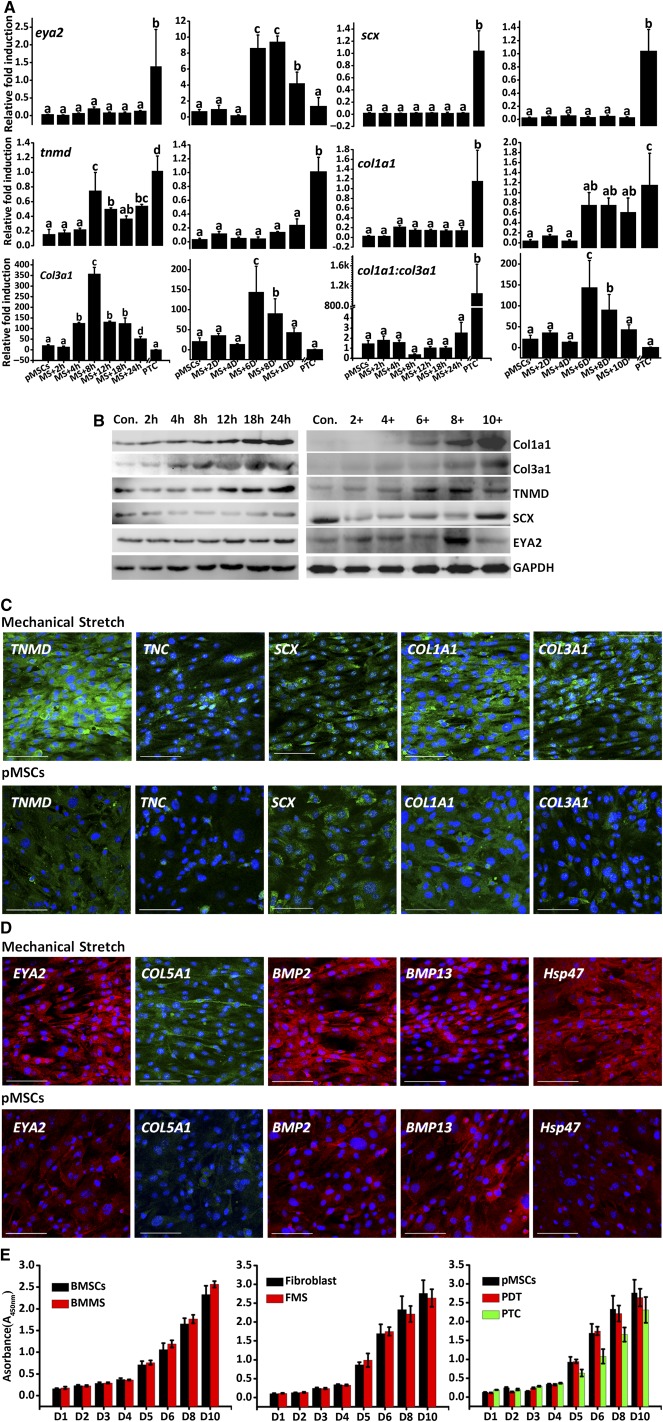
Tenogenic induction and investigation. **(A):** q‐PCR analysis of the expression of tenocyte‐specific markers in pMSCs following exposure to 0.1 Hz/10% mechanical stretch for 24 hours and 10 days. Graph bars with letters on top represent statistically significant results (*p* < .05) based on Newman‐Keuls post hoc one‐way ANOVA, whereas bars with the same letter correspond to results that show no statistically significant differences. In the case where two letters are present on top of the bars in Figure, each letter should be compared separately with the letters of other bars to determine whether the results show statistically significant differences. **(B):** Western blot analysis of the expression of tenocyte‐specific markers in pMSCs following exposure to 0.1 Hz/10% mechanical stretch for 24 hours and 10 days. **(C):** Immunocytochemistry staining for collagen biosynthesis, tenocyte‐specific markers, tenocyte healing. Antibody staining (green and red), with DAPI nuclear staining (blue). Scale bars = 100 μm. **(D):** Growth kinetics of pMSCs, BMSCs, fibroblasts, PDT, BMMS, FMS, and PTC. The results shown are means ± SDs from three individual experiments. Abbreviations: ANOVA, analysis of variance; BMMS, BMSCs after 10 days of stretch; BMSCs, bone marrow stem cells; DAPI, 4′,6‐diamidino‐2‐phenylindole; FMS, pMSCs after 10 days of stretch; GAPDH, glyceraldehyde 3‐phosphate dehydrogenase; PDT, parthenogenetic stem cell‐derived tenocytes; pMSCs, parthenogenetic mesenchymal stem cells; PTC, patellar tenocytes; q‐PCR, quantitative polymerase chain reaction.

To determine whether pMSCs were tenogenic, we used a mechanical stimulation assay. Dermal fibroblasts, bone marrow stem cells, and patellar tenocytes (PTCs) acted as controls ( supplemental online Fig. 18). After exposure to 10% mechanical stretching for 8 hours or 10 days, the expression levels of the genes *COL1A1*, *COL3A1*, *TNMD*, and *EYA2* exhibited an approximate 18‐fold upregulation. No substantial upregulation of *SCX* was observed (Fig. 2A; supplemental online Figs. 19, 20). In addition, the apex ratios of *COL1A1:3A1* for both groups were 9.81 ± 0.59 (24 hours) versus 19.64 ± 3.18 (10D) (MSC group; supplemental online Fig. 19), 6.77 ± 0.18 (24 hours) versus 38.23 ± 3.08 (10D) (fibroblast group; supplemental online Fig. 20), and 2.53 ± 0.12 (24 hours) versus 8.07 ± 1.46 (10D) (pMSCs group; Figure 2A), suggesting that relatively longer mechanical stimulation will assist cell in differentiation and maturation. The increased ratio of *COL1A1:3A1* was achieved early on for fibroblasts and BMSCs, whereas pMSCs reached their apex at 10 days. Western blot analysis also showed that expression of the tenogenic markers *COL1A1*, *COL3A1*, *TNMD*, and *EYA2* was increased after mechanical stimulation for 24 hours (Fig. 2B).

Immunocytochemistry results confirmed that mechanical stretching significantly enhanced collagen biosynthesis (as shown by increased expression of *COL1A1*, *COL3A1*, *COL5A1*, and *HSP47*), induced tenocyte formation, and promoted tenocyte phenotype (as shown by increased expression of *BMP2*, *BMP13*, *TNMD*, *EYA2*, and *TNC*; Figure 2C). In addition, mechanical stretch had no significant influence on cell proliferation at the end‐point of loading (Fig. 2D).

Taken together, these results provided evidence that mechanical stimulation could enhance the tenogenic phenotype of cells.

### PDTs Could Proliferate on PLGA Scaffolds

**Figure 3:**
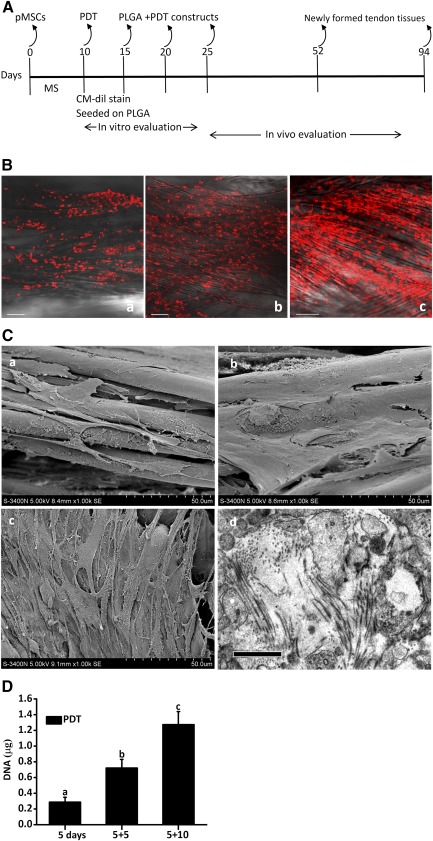
Tissue‐engineered tendon regeneration. **(A):** Strategy for building tendon‐like tissue in vitro and in vivo. **(B):** Confocal laser microscope observations of cell adhesion and growth on PLGA at 5 **(Ba)**, 10 **(Bb)**, and 15 days **(Bc)**. Scale bars = 100 μm. **(C):** SEM images of the cell‐scaffold constructs at 5 **(Ca)**, 10 **(Cb)**, and 15 days **(Cc)**. TEM images showing collagen fiber deposition in the cell‐scaffold construct at 15 days **(Cd)**. Scale bars = 1 μm. **(D):** PDTs proliferation on PLGA scaffold. a, b, and c represent statistical groupings with differences between each other of *p* < .05. Abbreviations: MS, mechanical stretch, PDT, parthenogenetic stem cell‐derived tenocyte; PLGA, poly(lactic‐co‐glycolic) acid; pMSC, parthenogenetic mesenchymal stem cell; SEM, scanning electron microscopy; TEM, transmission electron microscopy.

At 10 days after mechanical loading, PDTs were seeded on PLGA fibers and cultured for 15 days in vitro. CM‐Dil staining and SEM observations indicated that PDTs adhered to and proliferated well on PLGA fibers, as shown in Figures 3B and 3C. PDTs bridged the fibers, covered the surface of the scaffolds and began to secrete fine collagen fibrils 15 days after seeding (Fig. 3C). DNA content increased steadily during in vitro incubation, further demonstrating cell proliferation on the scaffold. These results indicated that the PLGA scaffold was suitable for PDTs cultivation (Fig. 3D).

### PDTs Regenerated Tendon‐Like Tissue In Vivo

**Figure 4:**
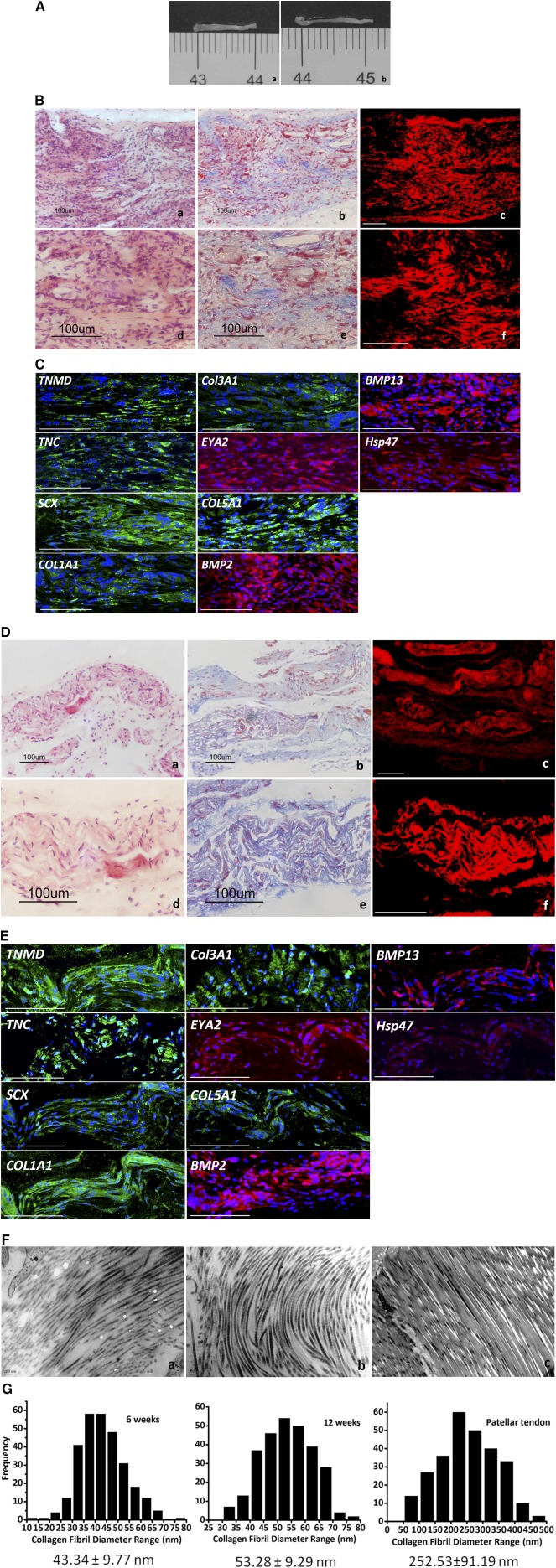
Analysis of newly formed tendon. **(A):** Gross inspection of the in vivo engineered new tendon at 6 **(Aa)** and 12 weeks **(Ab)**. **(B):** Histology of the in vivo engineered new tendon at 6 weeks. [**(Ba, Bd):** H&E staining; **(Bb, Be):** Masson's trichrome staining]. **(Bc, Bf):** Laser confocal microscope analysis; the red fluorescent signal indicates survival of implanted cells after 6 weeks. **(C):** Immunohistochemistry staining for tendon‐related markers. Antibody staining (green or red), with DAPI nuclear staining (blue). **(D):** Histology of the in vivo engineered new tendon tissue at 12 weeks. **(Da, Dd):** H&E staining; **(Db, De):** Masson's trichrome staining. **(Dc, Df):** Laser confocal microscope analysis; the red fluorescent signal indicates survival of implanted cells. **(E):** Immunohistochemistry staining for tendon‐related markers. Antibody staining (green or red), with DAPI nuclear staining (blue). Scale bars = 100 μm. **(F):** TEM images of the in vivo engineered new tendon tissues at 6 and 12 weeks. **(G):** Changes in collagen fibril diameter during in vivo implantation. Sample at 12 weeks had larger collagen fibers (53.28 ± 9.29 nm vs. 43.34 ± 9.77 nm). The fibrils from the 6‐week‐old mouse patellar tendons group were 252.53 ± 91.19 nm. Abbreviations: H&E, hematoxylin and eosin; DAPI, 4′,6‐diamidino‐2‐phenylindole; TEM, transmission electron microscopy.

Next, we examined the ability of PDTs to regenerate tendon‐like tissue in vivo. All animals survived throughout the experiment. The animals were sacrificed at weeks 6 and 12 after implantation to harvest specimens. Gross inspection showed that the newly formed tendon had a smooth and glistening white surface (Fig. 4A). Histology results showed that there was no teratoma formation after implantation. At 6 weeks, H&E staining revealed the tissue containing a large number of spindle‐shaped cells, which were situated along the axis of PLGA fibers. Masson's trichrome staining revealed some positively stained area, indicating collagen deposition in the construct. PLGA scaffolds were partially degraded (Fig. 4B). Transmission electron microscopy (TEM) observations showed that some fine collagen fibers, having an average diameter of 43.34 ± 9.77 nm, formed in the construct (Fig. 4A, 4G).

In contrast, the number of cells in newly formed tendon tissues was significantly lower at 12 weeks than at 6 weeks postoperation (Fig. 4C). H&E and Masson's trichrome staining revealed a structure of longitudinally aligned fibers and cells with increased matrix deposition. Typical crimp‐patterned collagen fibers with proper cell density could be observed, and most of the PLGA scaffolds were degraded. TEM observations further showed the formation of wave‐like collagen fibers, having an average diameter of 53.28 ± 9.29 nm, in the specimen (Fig. 4B, 4G).

The red fluorescent signal of CM‐Dil‐labeled cells was distributed in the newly formed tissue (Fig. 4B, 4D). Immunohistochemistry staining confirmed that the engineered tendon sustained expression of tendon‐specific markers, including *COL1A1*, *COL3A1*, *COL5A1*, *TNC*, *TNMD*, *SCX*, *HSP47*, *EYA2*, *BMP2*, and *BMP13* (Fig. 4C, 4E).

## Discussion

An optimal cell source for tendon regeneration should possess high proliferative and biosynthetic activity. In this study, we examined the capacity of pSCs to maintain basic biological characteristics of biparental stem cells (J1 cells). We found that pSCs could be differentiated into pMSCs, which had the potential to differentiate into three mesenchymal lineages. With in vitro cyclical mechanical stretch, we then directed pMSCs to differentiate into PDTs, which expressed tenocyte‐specific markers. Finally, we successfully engineered tendon tissue in vivo. These findings, together with the fundamental advantages (technical, ethical, and immunological) of pSC application, demonstrated that pSCs may represent an efficient and practical strategy for tendon healing and regeneration.

It would be ideal to repair injured tendon with autologous cells. However, treatment generally occurs under conditions of acute injury. Therefore, because application of autologous cells requires a time‐consuming expansion process (within weeks or months) to obtain sufficient numbers of tenogenic cells, autologous transplantation may not be practical 31. Furthermore, the fundamental limitations of the available autologous cells, including tenocytes, BMSCs, and fibroblasts, will significantly influence the therapeutic effect. pSCs, which have ethical and technical (no genetic manipulation required) advantages, may be applicable due to their widely haploidentical genomes, which would be expected to increase cell immunotolerance for allogeneic transplantation and provide a realistic source for cell‐based tendon repair.

Both pSCs and J1 cells expressed pluripotent markers, formed colonies, proliferated actively, and generated EBs effectively ( supplemental online Figs. 1–7). pSCs had relatively lower saturation densities and proliferation rates than J1 cells, consistent with previous studies 32. The expression of maternally imprinted genes (i.e., *IGF2*) is unequal expression in pSCs from various species and may influence cell proliferation and growth patterns 33 34 35. Whereas pSCs only exhibited a dot‐patterned colony shape, J1 cells exhibited dot‐ and ring‐patterned colony shapes. We hypothesize that this may be due to the higher expression levels of vitronectin (which is involved in the spreading of cells and stabilization of cell adhesion), or decreased E‐cadherin rapidly in J1, suggesting an EMT that exhibited the differentiation of early commitment to mesenchymal fates and may increase cell migration 36.

The development of mesodermal lineages is compromised in parthenotes 35, 37. Our results showed that there were no obvious differences in differentiation potential between pSCs and J1 cells ( supplemental online Figs. 7–11). Moreover, pSCs could spontaneously differentiate into three germ lines in EBs and their outgrowths, which may be particularly attractive for cell‐based therapy. As demonstrated by Q‐PCR, pSC derivatives expressed various mesodermal lineages markers, such as lateral plate/extraembryonic, paraxial/myogenic, and intermediate mesoderm. Even the alteration trends of most detective gene were the same. This was somewhat unexpected but attractive. In addition, the formation mesodermal lineages components were observed (Figs. 1, 2), and pSCs were shown to form mature cartilage, muscle, and osseous tissue ( supplemental online Fig. 11). These results were consistent with the report by Didié et al. and Liu, who reported that murine PSCs and ESCs had similar fundamental properties, despite notable differences in genetic (allelic variability) and epigenetic (differential imprinting) characteristics 30, 38, provided a solid basis for subsequent pSC differentiation into mesodermal lineages, including tenocytes.

A number of factors have been identified as important in maintaining the tenogenic phenotype, including ECM, growth factors, mechanical stimulation, and oxygen tension. The mechanical niches have been identified with in a variety of cell 39 40 41. We found that mechanical stretch was efficient in improving the tenogenic phenotype of pMSCs, BMSCs, and fibroblast. A combination of Q‐PCR, immunocytochemistry, and Western blot analyses showed that mechanical stimulation was sufficient in upregulating tenocyte‐specific markers at both the mRNA and protein level; these effects were considered important for the maturation of tenocytes (Fig. 2, supplemental online Figs. 19, 20). Cell responses to mechanical stimulation varied among the different types of cells. Future studies should examine whether the differences in mechanical stretch patterns are associated with changes in the efficiency of differentiation into tenocytes.

Finally, we tested the applicability of PDTs in tendon regeneration. PLGA‐scaffold used in this study suited cellular attachment, growth, and ECM formation (Fig. 3). In vivo tests confirmed that the newly formed tissue possessed the similar structure and ECM components of primary tendons. Histological observation showed that wave‐like collagen fibers formed in the specimen, and the implanted cells remained viable in vivo for up to 12 weeks. No teratoma formation could be observed in any specimen (Fig. 4). Compared with the native patellar tendon, the newly formed tendons tissue in the current experiment exhibited looser structures (Fig. 4D) and smaller collagen fiber diameters (Fig. 4G), as evidenced by Masson's trichrome staining and TEM analysis. The less ideal structure of the newly formed tendons may be partially due to the lack of physiological mechanical stimulation in subcutaneous sites.

In the current experiment, we show that PDTs could form tendon‐like tissue in ectopic site in vivo. Human PDTs may have implications for the treatment of tendon injury by local delivery, or for the treatment of tendon defect with the combination with scaffold. Therefore, the future goal of our study should be to further investigate the tendon regeneration potential of PDTs in situ in immunocompetent animal models.

## Conclusion

Our study successfully demonstrated that pSCs function as a unique cell type capable of differentiation into tenocytes. pSCs showed almost the same characteristics as biparental stem cells. Under in vitro cyclical mechanical stretch, pMSCs differentiated and formed tenocytes. Moreover, PDTs seeded on PLGA scaffolds formed neo‐tendon tissues in mice. Our studies of pSC properties showed that these cells are attractive alternatives and that may represent a novel tool for tissue engineering.

## Author Contributions

Wei Liu: conception and design, manuscript writing, data analysis and interpretation; L.Y., Wenguang Liu, Y.R., M.S., and Q.W.: provision of study material or patients; X.Y. and J.C.: collection and/or assembly of data; F.C.: conception and design, financial support, final approval of manuscript.

## Disclosure of Potential Conflicts of Interest

The authors indicated no potential conflicts of interest.

## Supporting information

Supporting Information

Supporting Information
