# Supplementary material for: Directing the Differentiation of Parthenogenetic Stem Cells into Tenocytes for Tissue‐Engineered Tendon Regeneration
Source: Stem Cells Transl Med. 2016 Aug 18;6(1):196–208. doi: 10.5966/sctm.2015-0334 (PMC5442735; doi:10.5966/sctm.2015-0334)
Supplement: Supplementary file 1 — Supporting Information [file SCT3-6-196-s001.pdf]

Supplemental Information

Materials and methods

Cell isolation and culture

pSCs (C57BL/6 strain) and ESCs (J1; 129S4/SvJae strain) were cultured on mitotically inactivated mouse embryonic fibroblasts and maintained in Esgro Complete Plus (Millipore, Billerica, MA, USA). Patellar tendons from 3~6-week-old mice were dissected as follows. First, tendon tissues were cut into small pieces, and digested with 1 mg/mL collagenase type I (Millipore) in phosphate-buffered saline (PBS) for 3–4 h at 37°C. Single-cell suspensions were cultured in Dulbecco's modified Eagle's medium (DMEM; Sigma, St. Louis, MO, USA), supplemented with 20% fetal bovine serum (FBS; Gibco, Grand Island, NY, USA). We also isolated and cultured dermal fibroblasts and BMSCs from the same mouse.

TEM

For TEM, the cells were trypsinized, rinsed, and centrifuged. Then, the cells were fixed in fixative solution (2.5% glutaraldehyde and 2% formaldehyde) embedded in epoxy resin. The prepared samples were sectioned to 70–90-nm thicknesses. Ultrathin sections were stained in 3% aqueous uranyl acetate and then in Sato triple lead stain prior to examination using an FEI CM12 Electron Microscope (FEI Co., Hillsboro, OR, USA).

Immunocytochemistry

pSCs, J1 cells, EBs, EB outgrowths, and pMSCs were cultured on glass coverslips coated with gelatin and then fixed with 4% paraformaldehyde in PBS for 1 h at 4°C. Cells were then permeabilized for 8-15 min in PBS containing 0.5% Triton-X 100, blocked for 2 h in PBS containing 10% bovine serum albumin (BSA), and immunostained with primary antibodies overnight at 4°C. Primary antibodies used were as follows: anti-Nanog, anti-OCT4, anti-SSEA-1, anti-Vimentin, anti-CD34, anti-Sca-1, anti-N-cadherin, anti-E-cadherin, anti-Nt5e, anti-α-actin (Santa Cruz Biotechnology, Santa Cruz, CA, USA), anti-MHC (myosin heavy chain), and anti-Myog

(Myogenin) (Abcam, Cambridge, MA, USA). After two washes with PBS, the slides was incubated with suitable Alexa Fluor 488- or Alexa Fluor 594-labeled secondary antibodies (Invitrogen) in the dark for 1 h at 37°C. After two washes, nuclei were counterstained with 4′, 6-diamidino-2-phenylindole (DAPI; Invitrogen). Images were taken with a laser confocal microscope (FV1000; Olympus Corporation, Tokyo, Japan). For negative controls, isotype-matched negative control antibodies (Santa Cruz Biotechnology) were used under the same conditions.

Colony formation assay

The cells were dissociated with Accutase (Millipore) and resuspended to obtain single-cell suspensions of pSCs and J1. After that, the cells were counted and seeded into six well plates at four densities (100, 250, 1000 and 1500 cells/well) to form colonies in Esgro Complete Plus medium in triplicate for each cell density without feeder layer. After 5 days, the formed colonies were stained with 1% crystal violet and counted (100, 250 cells/well).

WST-1 cell proliferation assay

Cell viability was analyzed using 4-[3-(4Iodophenyl)-2-(4-nitrophenyl)-2H-5-tetrazolio]-1, 3-benzene disulfonate (WST-1) assays. For this procedure, pSCs and J1 cells were seeded in 96-well plates at different cell densities from 1000 to 2000 cells/100 µL in Esgro Complete Plus medium in quintuplicate for each cell density. A 10-µL volume of cell proliferation reagent WST-1 (Roche, Germany) was added to each well, and plates were incubated at 37°C/5% CO_2 in a humidified incubator for up to 60 h. The absorbance was measured every 12 h in a microplate reader (BioTek Synergy HT, Winooski, VT) at 450 nm.

AP activity

Cellular AP activity was detected using an AP Kit (Jianchen, Nanjing, China) following the manufacturer's instructions.

Light microscopy

Cells were visualized using a phase contrast microscope (TE2000-U; Nikon Inc.) throughout

culture.

EB formation

The pSCs and J1 cells were dissociated with Accutase (Millipore) and resuspended in cell growth medium (CGM; containing DMEM [Sigma] supplemented with 1% non-essential amino acids [Millipore], 1% 2-mercaptoethanol [Sigma], 50 U/mL penicillin [Sigma], 50 mg/mL streptomycin [Sigma], 2 mM L-glutamine [Sigma], and 20% FBS [Gibco]). To explore whether pSCs could form EBs and to examine the EB formation efficiencies of pSCs and J1 cells, we used limiting dilution to place one cell into separate wells of ultra-low attachment 96-well plates (Fisher Scientific, Ottawa, Ontario). Each well was examined to verify the number of cells 2 day after plating. The tightly packed spheres were identified as EBs and counted 5 days later.

TUNEL assay

The 5-day EBs were fixed in 4% paraformaldehyde for 24 h, dehydrated, embedded in paraffin, and sectioned. Representative sections were processed for H&E staining and TUNEL assay. Apoptotic cells were detected with an *In Situ* Cell Death Detection kit (Roche, Germany) according to the manufacturer's protocol. Nuclei were counterstained with DAPI. The numbers of apoptotic nuclei (TUNEL staining) and total numbers of nuclei were determined by counting. To quantitatively analyze the apoptotic nuclei, about 10–12 EBs were randomly selected and counted from each group.

Spontaneous differentiation in adherent culture

The 5-day EBs were plated onto dishes coated with 0.1% gelatin (Sigma) to evaluate their developmental potential by monitoring the expression of specific markers. EB outgrowths were allowed to spontaneously differentiate in CGM for 28 days with medium changes every 3–4 days.

Gene expression analysis by Q-PCR

Total RNA was extracted from the cells using an RNA Isolation Reagent (Takara Bio Inc., Japan) according to the manufacturer's protocol. The extracted RNA was quantified using a

GeneQuant pro (GE Healthcare, USA). A RevertAid First Strand cDNA Synthesis Kit (Thermo Fisher Scientific, Waltham, MA, USA) was used to convert the RNA template into cDNA. Q-PCR was performed using a Bio-Rad Q-PCR system (Bio-Rad, Hercules, CA, USA). The relative levels of gene expression were conducted, using the comparative $\Delta\Delta C_T$ method, with β-actin as an internal control and normalized to the control group.

The Primers sequence for Q-PCR

Gene	Primers	Product
igf2r	CTCACCTACTACGACGGAATGATC	156bp
	GGTAGAGTTGTCCTCTTCCTGATATTC	
$p57^{kip2}$	CAGCGATACCTTCCCAGTGATAG	155bp
	CATCCTGCTGGAAGTTGAAGTCC	
igf2	GCGGCTTCTACTTCAGCAG	71bp
	GCAGCACTCTTCCACGATG	
vtn	CGAAGGCTTCAGTGGCATAC	179bp
	GCTCAAACACGGCTGACA	
itgb1	TGGTCAGCAACGCATATCTG	118bp
	CACCAGCAGTCGTGTTACA	
snai1	GACCTGTGGAAAGGCCTTCTCTAGG	170bp
	CCTGGCACTGGTATCTCTTCACATC	
slug	GGGAGCATACAGCCCTATTACTG	146bp
	CCTTGGATGAAGTGTCAGAGGAA	
n-cadherin	CGCAGTCTTACCGAAGGATGTG	170bp
	GCTCTGCAGTGAGAGGGAAG	
e-cadherin	CCACCGATGCAGACGATGA	183bp
	CCTGAACCACCAGAGTGTATGTAG	
hand1	GCTACGCACATCATCACCATCATC	125bp
	CAGCAGCCAGCTCTGGAAGTAAG	
gata2	GCCAAAAGAGAGACTGGAGGAAGGG	82bp
	ACACCTCCCACCTTTTAGTCACTCTG	
meox1	CAGTCAAAATGTTCAGCATGGTAG	195bp
	AGAGGAAAATGTTGAATGGAAACTTTA	
myf5	CCTGAAGAAGGTCAACCAAGCTTTC	158bp
	GGCTGTAATAGTTCTCCACCTGTTC	
pax3	CTCTGAACCTGATTTACCGCTGAA	124bp
	CCTGGTGTAAATGTCTGGGTAGTG	

pax7	CCTCAGGTCATGAGCATCCTTAGC	192bp
	GTAGGTGGGTGGGCAGTAAGACTG	
chrd	GGTGCAAGTGGTAGGTACAGGTAG	182bp
	GCTCGTTCTGTAGCAGCATATGAG	
shh	CCCAATTACAACCCCGACATCATATTTAAG	196bp
	CCTCATAGTGTAGAGACTCCTCTGAATG	
pax2	CTGTCCCTAATGGAGACTCCCAGA	162bp
	CCTGTTCTGATTTGATGTGCTCTGATG	
osr1	GCCACTTCACTAAGTCCTATAACCTAC	169bp
	TTCCCACACTCTTGACACTTGAAA	
pax8	CAAGGTGGTGGAGAAGATAGGAGACTA	140bp
	GGATGATTCTGTTGATGGAGCTGACA	
α-[illegible]	CCCTCATCCTCCTGCTACATTTC	145bp
	GGAACAAACTGGGTAAAGGTGATG	
drd2	GACTCAACAACACAGACCAGAATG	138bp
	GCTTGCGGAGAACGATGTAG	
gata4	CTCCATGTCCCAGACATTCAGTAC	109bp
	CTGAGTGACAGGAGATGCATAGC	
fofx1	CCTACATCAAGCAACAGCCTCTG	157bp
	GACTGTGAGTGATACCGAGGGATG	
hand1	GCTACGCACATCATCACCATCATC	125bp
	CAGCAGCCAGCTCTGGAAGTAAG	
myod1	CTGAGCAAAGTGAATGAGGCCTTC	125bp
	AGAGCCTGCAGACCTTCGATGTAG	
notch1	GAGGCACCTGCCACAATGAGATC	189bp
	CACAGTTCTGTCCAGCAAAACCTG	
nanog	GCACTCAAGGACAGGTTTCAG	169bp
	GACCATTGCTAGTCTTCAACCAC	
oct3/4	GTGTGAGGTGGAGTCTGGAG	182bp
	AGCCTCATACTCTTCTCGTTGG	
ssea-1	GGTGGGTGTGCTGCTCTG	185bp
	GCCAGTCGTGGAGTTCCTTC	
tnmd	GCTGGATGAGAGAGGTTACTGTTG	144bp
	CAAGGCATGATGACACGACAGA	
scx	GTGCTGCTGGTGGGTGAG	180bp
	CTGGTTGCTGAGGCAGAAGG	
eya2	CAGTCCTCCACAGCCATGG	174bp
	GCTCAGGAACCCGCTCTG	

col1a1	GACTGCCTGGACCTCCTG	70bp
	GCAACACCATCAGCACCAG	
col3a1	GGATGGAGAGTCAGGAAGAC	76bp
	AGCTGGGCCTTTGATACC	

Gene	Primers	Product
alp	TCACGGCCATCCTATATGGTAAC	98bp
	CTGGTAGTTGTTGTGAGCGTAATC	
opn	CACACAGACTTGAGCATTCCAAA	77bp
	GGAACTTGCTTGACTATCGATCAC	
runx2	CCAGTCTTACCCCTCCTATCTGA	138bp
	GTGGCAGTGTCATCATCTGAAATAC	
ocn	CCATCTTTCTGCTCACTCTGCTG	136bp
	CGGAGTCTGTTCACTACCTTATTGC	
ibsp	GAGACGGCGATAGTTCCGAAGAG	132bp
	CCGAGAGTGTGGAAAGTGTGGAG	
bglap2	CAAGCAGGAGGGCAATAAGGTAG	133bp
	CTGGTCTGATAGCTCGTCACAAG	
col2a1	CCAGAACATCACCTACCACTGTAA	111bp
	GCCCTCATCTCTACATCATTGGA	
aggrecan	CCATGTGTGGGTGACAAAGACAG	92bp
	TCCACGTAGCAGTAGACATCATAGG	
c/ebp	CCGATGAGCAGTCACCTCCAGAG	169bp
	GGTCGATGTAGGCGCTGATGTCTA	
ap2	ACAGGAAGGTGAAGAGCATCATAA	143bp
	GGAAGTCACGCCTTTCATAACAC	

Gene	Primers	Product
tnmd	GCTGGATGAGAGAGGTTACTGTTG	144bp
	CAAGGCATGATGACACGACAGA	
scx	GTGCTGCTGGTGGGTGAG	180bp
	CTGGTTGCTGAGGCAGAAGG	
eya2	CAGTCCTCCACAGCCATGG	174bp
	GCTCAGGAACCCGCTCTG	
col1a1	GACTGCCTGGACCTCCTG	70bp
	GCAACACCATCAGCACCAG	
col3a1	GGATGGAGAGTCAGGAAGAC	76bp
	AGCTGGGCCTTTGATACC	

β-actin	GAGACAACATTGGCATGGCTTTG<br>CCTCAGCCACATTTGTAGAACTTTG	190bp

Teratoma assays

To evaluate whether pSCs possessed pluripotent differentiation capacity *in vivo*, the cells were dissociated with Accutase (Millipore), and 1×10^6 cells resuspended in 50 µL DMEM (Sigma) were injected subcutaneously into nude mice. After 3–4 weeks, the formed teratomas were harvested and fixed in 4% paraformaldehyde, dehydrated through a graded series of ethanol, embedded in paraffin, sectioned, stained with H&E, and observed.

Statistical analysis

The data are expressed as means ± SDs from three replicate experiments. Differences in pSCs, J1 cells, EBs, EB outgrowths, and osteogenic, chondrogenic, adipogenic lineages differentiation were assessed using unpaired Student's t-test in SPSS software (version 22.0). Data sets that involved more than two groups were assessed by one-way ANOVA followed by Newman–Keuls *post hoc* tests (see the Fig. S19 and Fig.S20). In the figures, the data with different superscript letters are significantly different based on *post hoc* ANOVA statistical analysis. Differences with *p* values of 0.05 were considered significant.

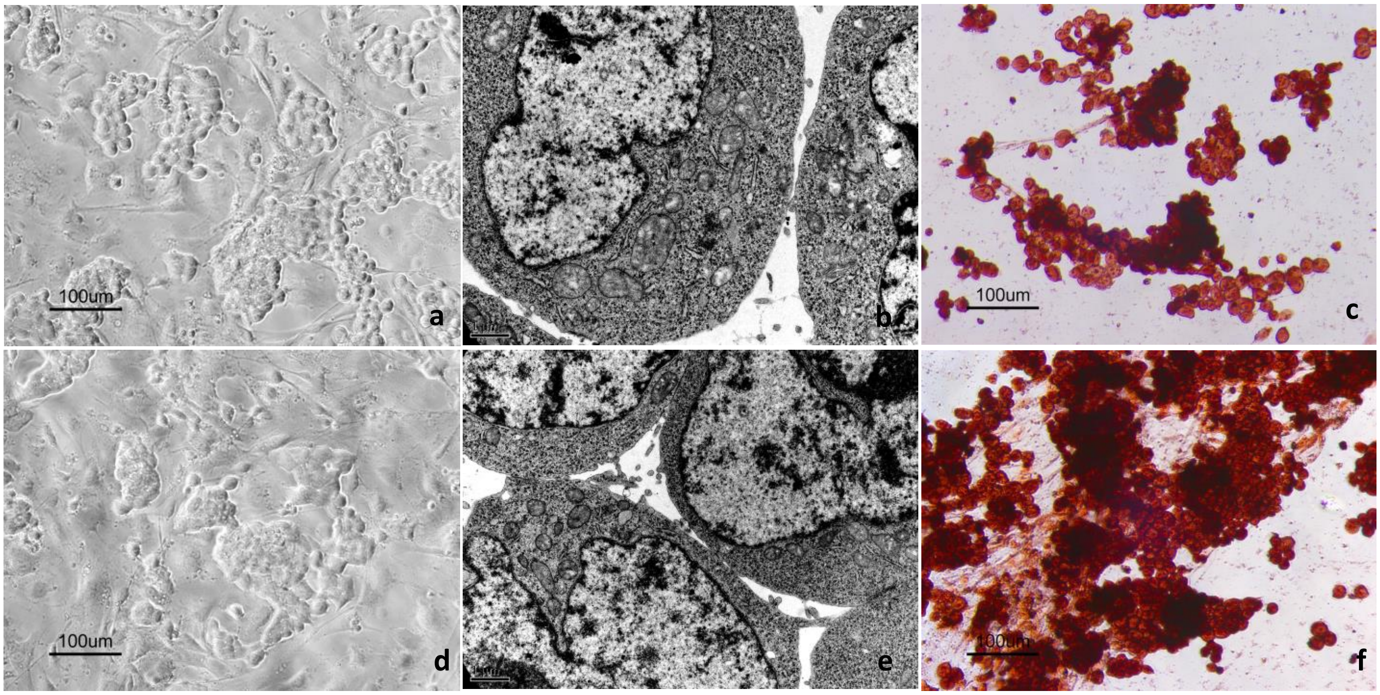

Figure S1. Morphological observations of pSCs (a) and J1 (d) under phase-contrast microscopy.

Bars = 100 μm. Transmission electron microscopy of the initial-stage of pSCs (b) and J1 cells (e).

Bars = 1μm. pSCs (c) and J1 cells (f) showed AP activity. Bars = 100 μm.

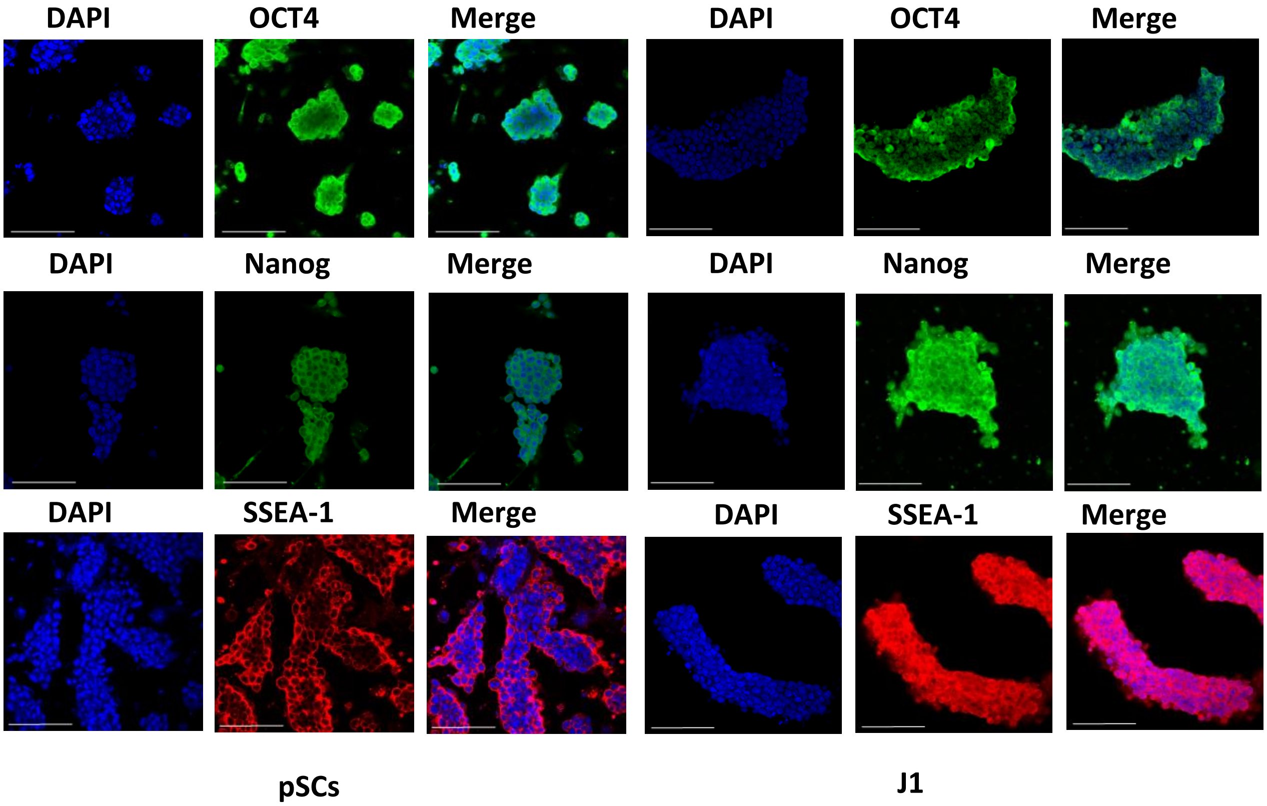

Figure S2. Immunocytochemistry staining of pluripotent markers in pSCs and J1, with DAPI nuclear staining (blue). Bars = 100 µm.

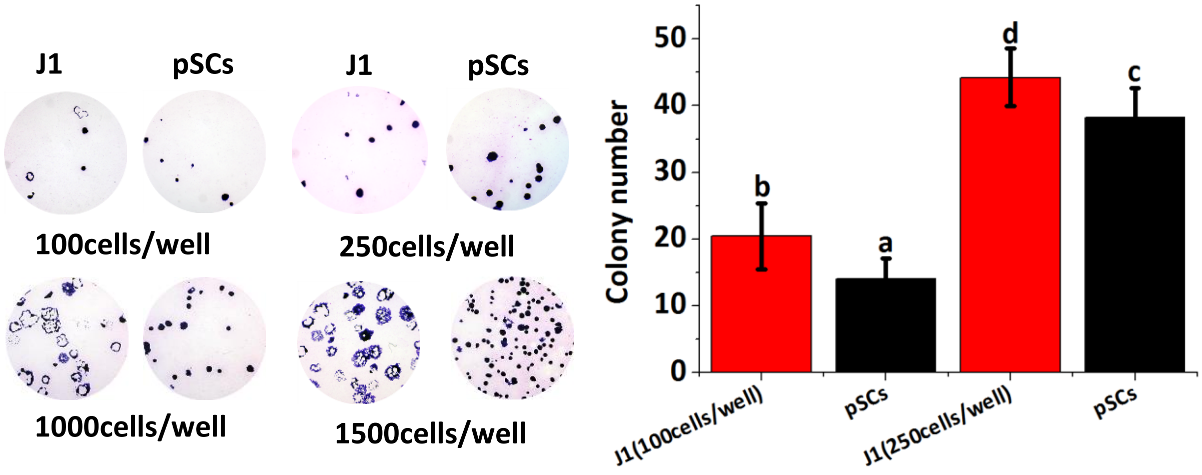

Figure S3. Colony-forming efficiencies of J1 cells and pSCs (20×magnification). Colonies were scored after 5 days. Graph showing the number of colonies formed. The results shown are means ± SDs from three wells. Graph bars with different letters on top represent statistically significant results ($p<0.05$) based on Newman–Keuls *post hoc* one-way ANOVA analysis, whereas bars with the same letter correspond to results that show no statistically significant differences. In the case where two letters are present on top of the bars in Figure, a, b, c and d represent statistical groupings with differences between each other of $p< 0.05$.

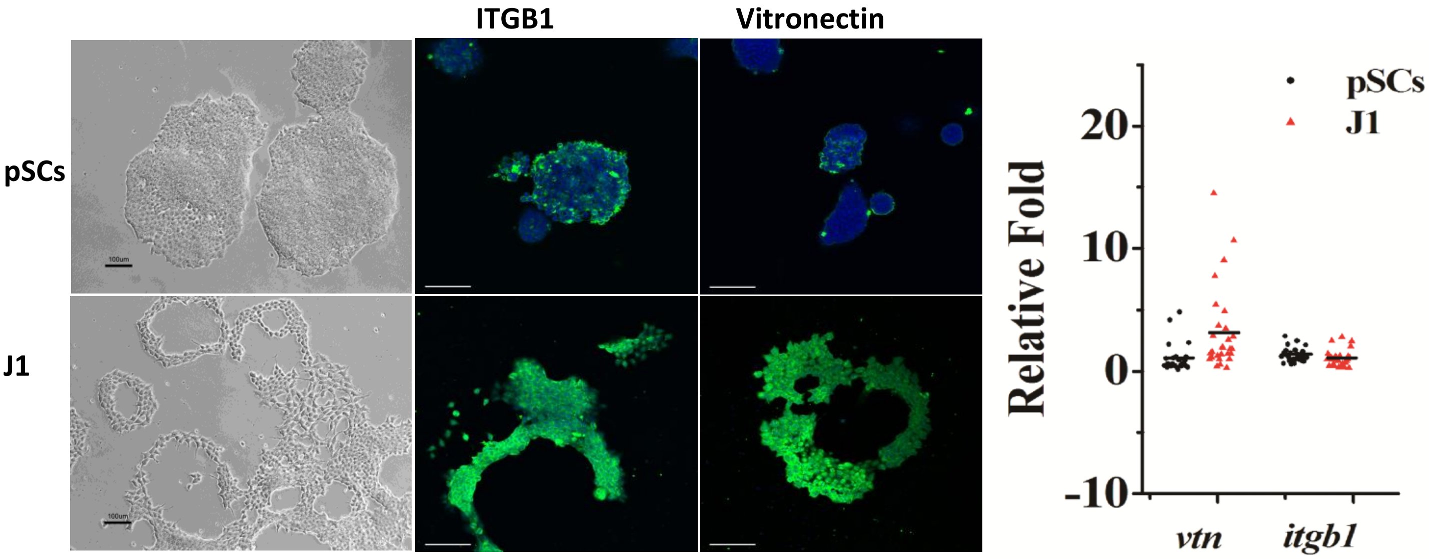

Figure S4. Morphological observations of pSCs and J1 under phase-contrast microscopy without feeder layer. Immunocytochemistry staining and Q-PCR analysis of molecules related to adhesion. Q-PCR analysis of *vtn* and *itgb1* expression during 10 continuous passages. Each dot on the graph represents the value of the sample. Horizontal lines are average values for all tested samples (*vtn* and *itgb1*: n = 30;). Bars = 100 μm

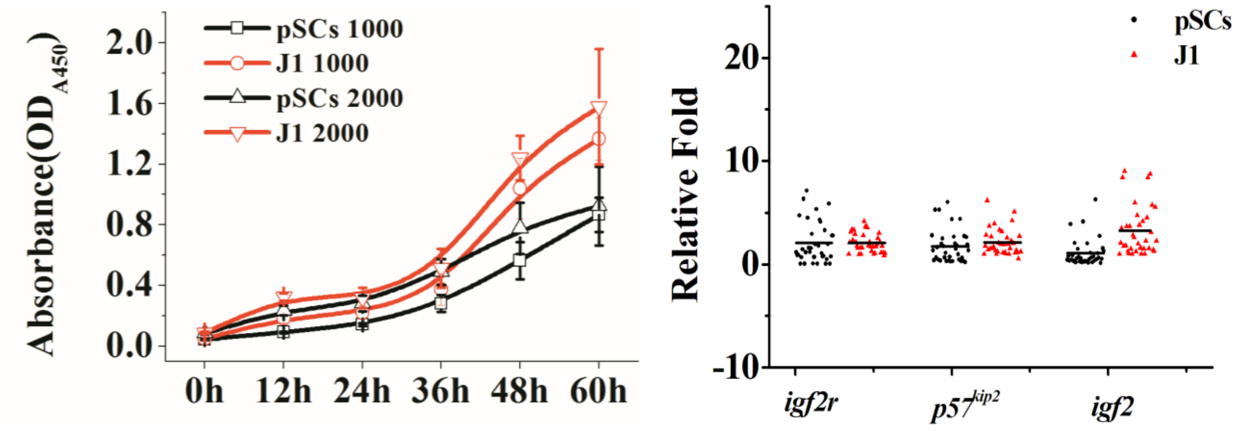

Figure S5. Growth kinetics of pSCs and J1 cells. Q-PCR analysis of *igf2r*, *igf2* and *p57*^{KIP2} expression during 12 continuous passages. Each dot on the graph represents the value of the sample. Horizontal lines are average values for all tested samples (*igf2r*, *igf2*, *p57*^{KIP2}: n = 36;).

Table 1S. EB formation efficiency

Experiment number(pSCs)	Plating cells	EBs	EB formation effiency(%)	Experiment number(J1)	Plating cells	EBs	EB formation effiency(%)
1	81	22	27.2	1	76	29	38.2
2	90	20	22.2	2	85	31	36.5
3	93	17	18.3	3	94	31	33.0
4	88	21	23.9	4	83	24	28.9
5	88	18	20.5	5	92	29	31.5
6	87	16	18.4	6	92	40	43.5
	87.8	19	21.7		87.0	30.7	35.3

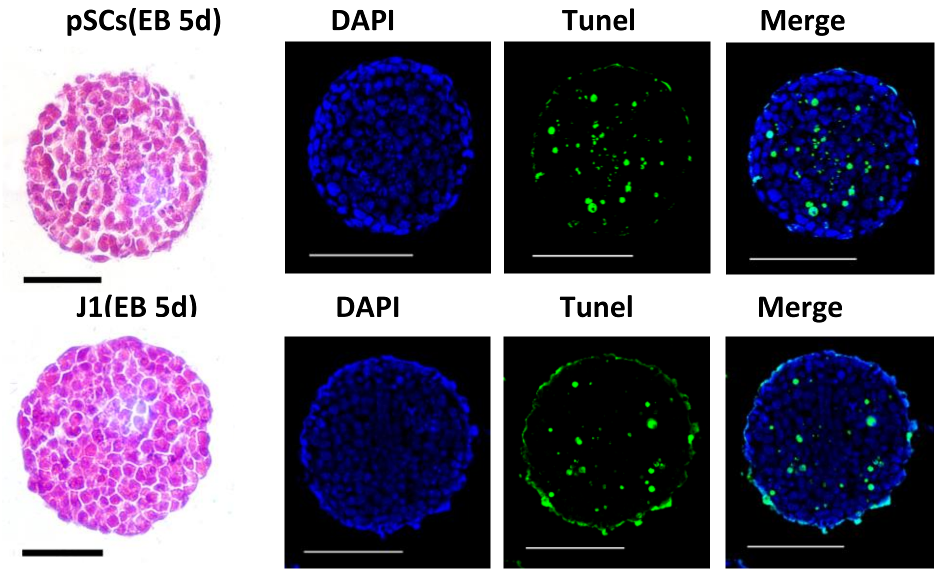

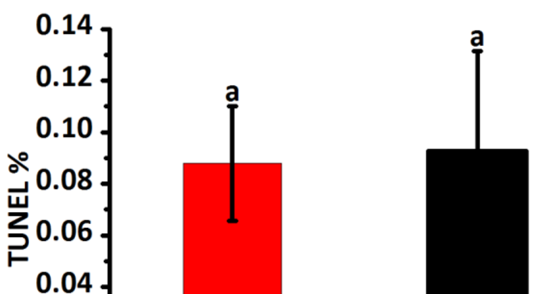

Figure S6. Embryoid body (EB) formation efficiency, H&E stain and TUNEL assays of EBs from pSCs and J1 cells at 5 days. Apoptotic cells stained green in EBs. Graph showing the apoptotic rate (number of apoptotic cells/number of total cells on the slide. The results shown are means ± SDs from 10 EBs (p = 0.728). Graph bars with different letters on top represent statistically significant results (p<0.05) based on Newman–Keuls *post hoc* one-way ANOVA analysis, whereas bars with the same letter correspond to results that show no statistically significant differences. In the case where two letters are present on top of the bars in Figure, each letter should be compared separately with the letters of other bars to determine whether the results show statistically significant differences.

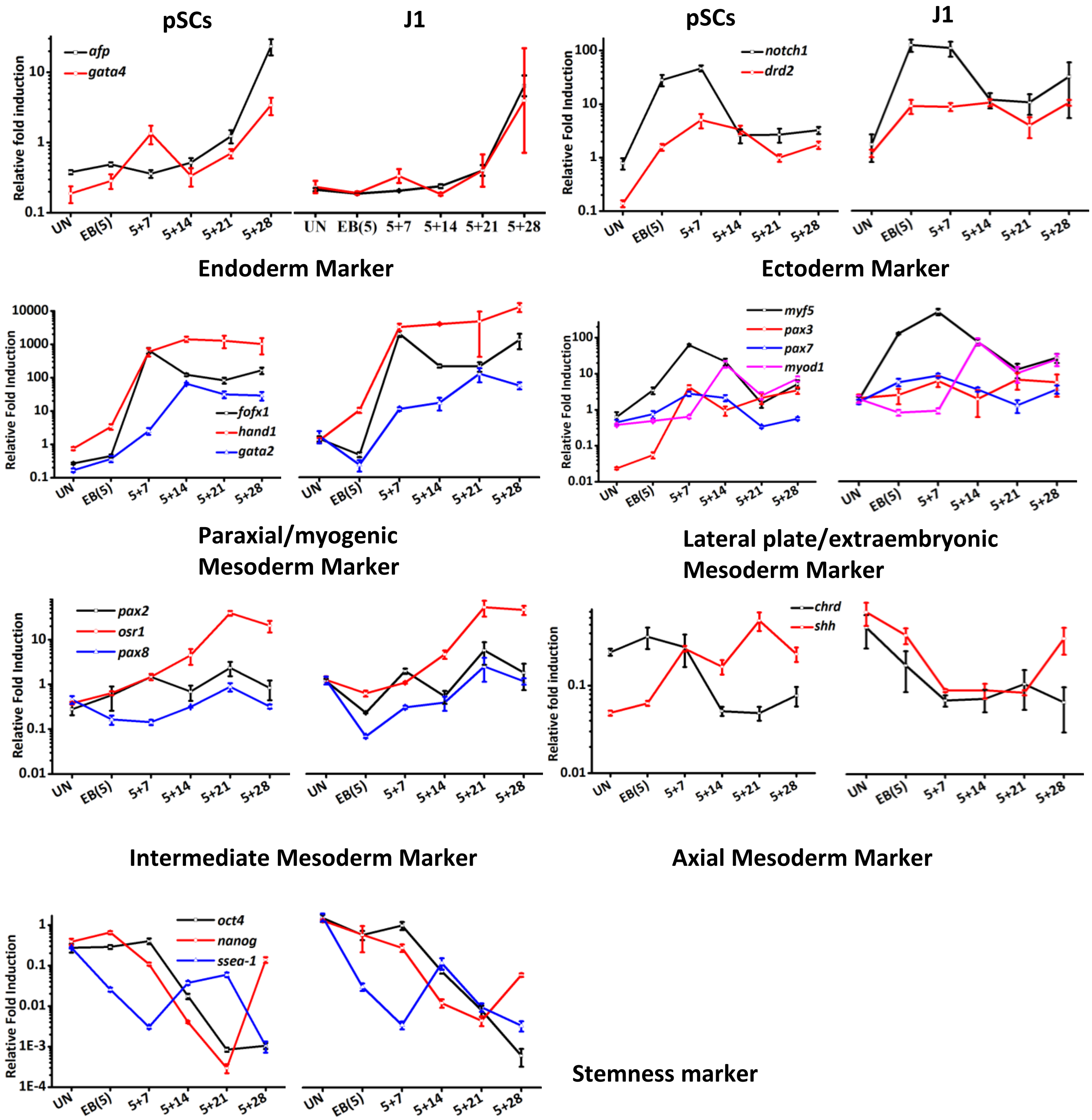

Figure S7. Q-PCR analysis of gene expression profiles related to the three germ layers of samples from pSCs and J1 cells. Initial-stage of pSCs and J1 cells (UN), Five-day EBs (5) and (+):

subsequent adhesion culture days. The results shown are means ± SDs from three individual experiments.

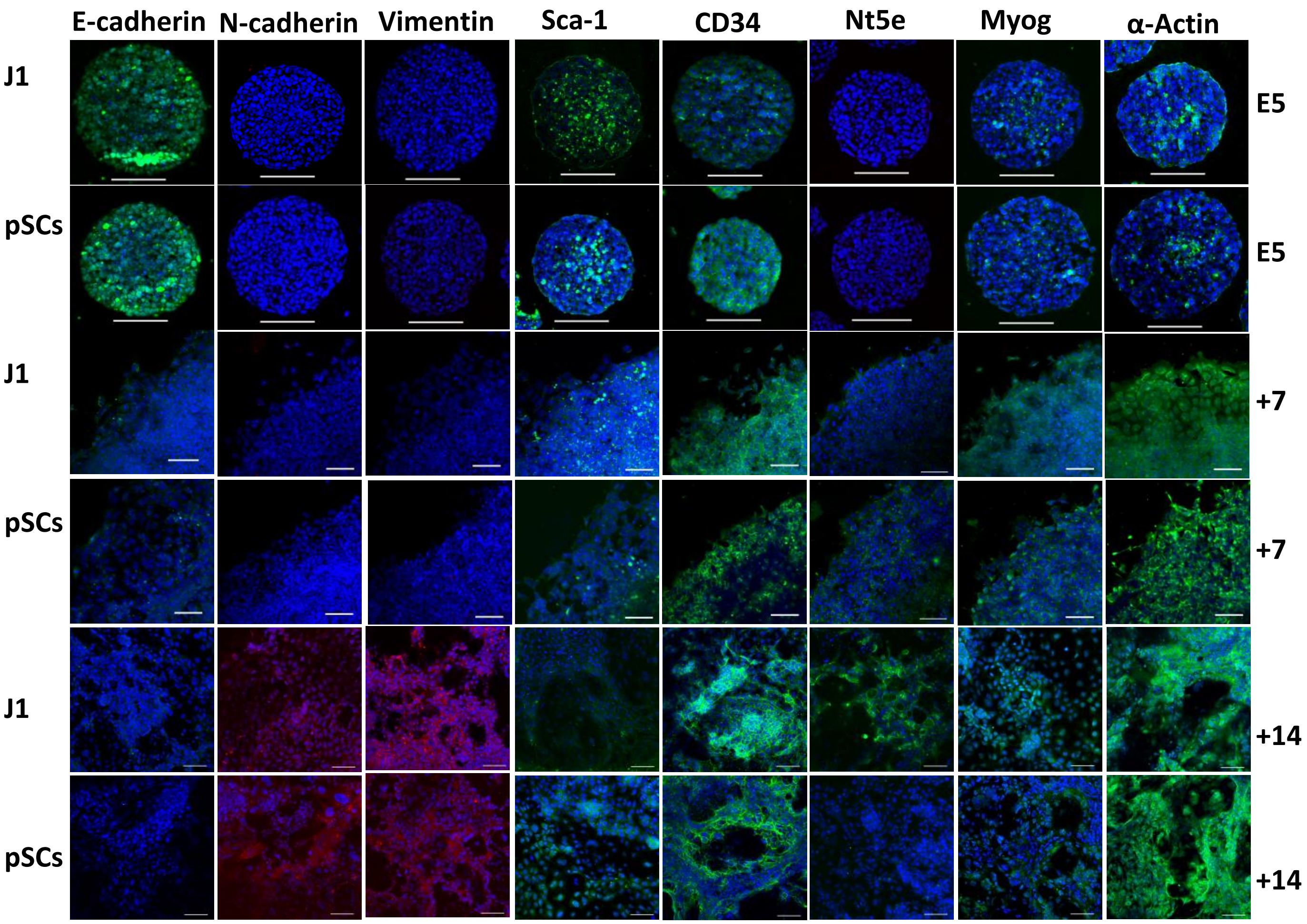

Figure S8. Immunocytochemistry staining of proteins related to the EMT-MET and mesodermal differentiation in 5-day EBs and (+): subsequent adhesion culture days. Antibody staining (green and red) with DAPI nuclear staining (blue). Bars = 100 μm.

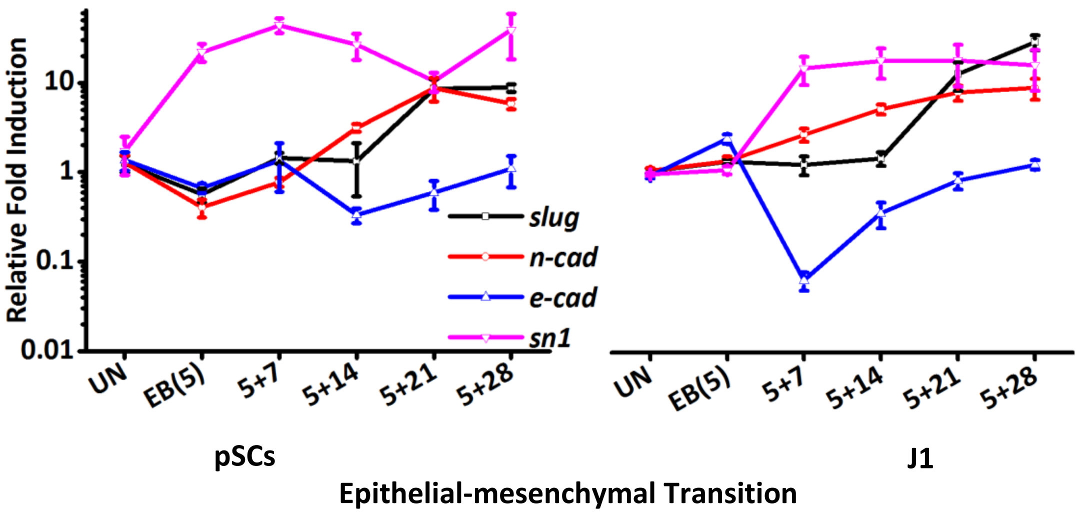

Figure S9. Q-PCR analysis of gene expression profiles related to EMT/MET markers in initial-stage of pSCs and J1 cells (UN), Five-day EBs (5) and (+): subsequent adhesion culture days. The results shown are means ± SDs from three to six individual experiments.

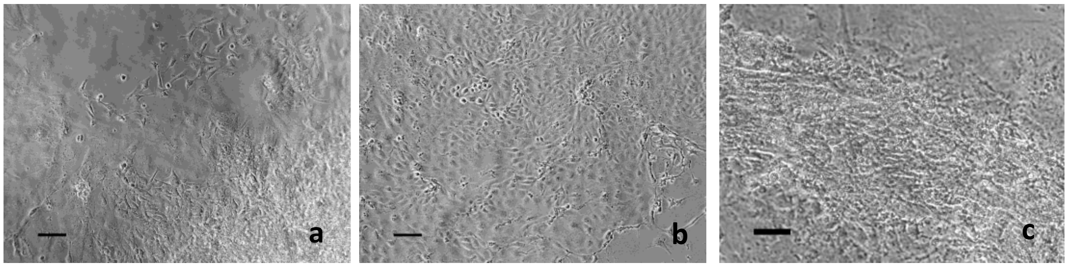

Figure S10. Outgrowth from EBs derived from pSCs resembled: a, fibroblast-like cells; b, epithelial-like cells; and c, beating cardiomyocytes. Bars = 100 µm.

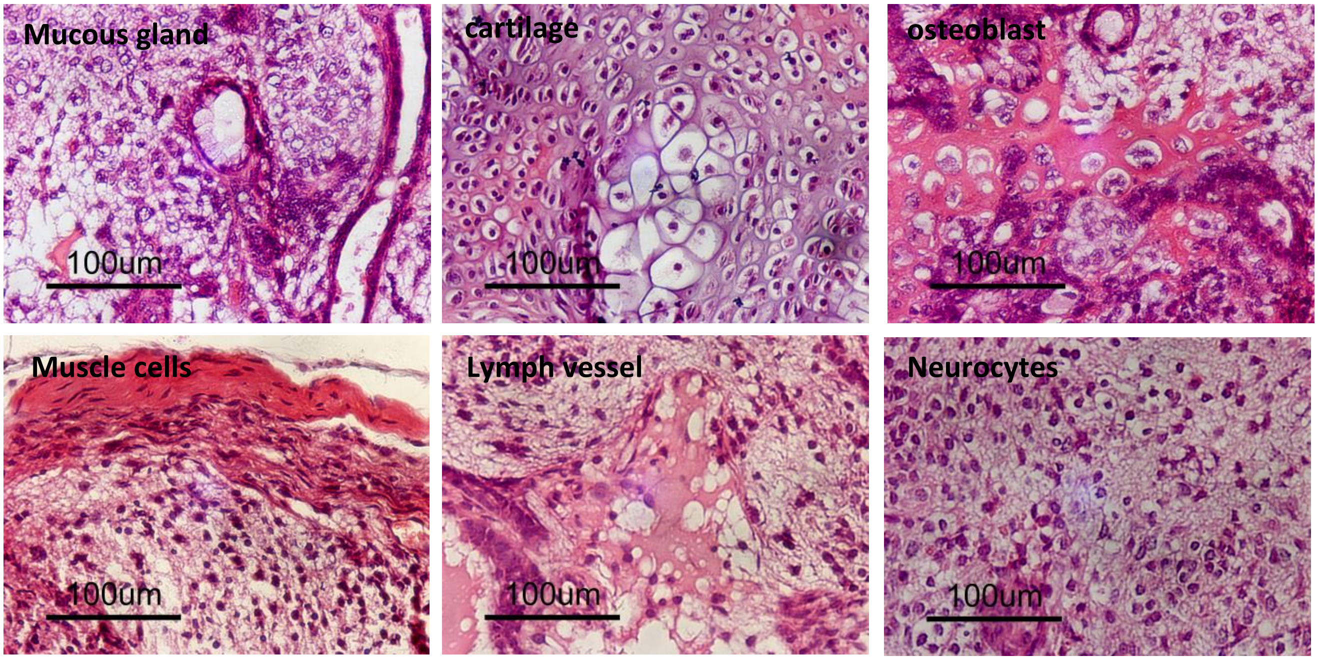

Figure S11.H&E staining of teratomas revealed that pSCs possessed the capacity to generate mucous glands, cartilage, osteoblasts, muscle cells, lymph vessels, and neurocytes *in vivo*. Bars = 100 µm.

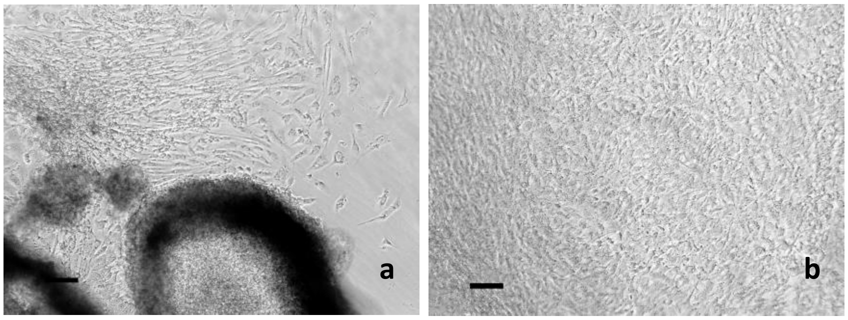

Figure S12. Generation of MSCs from J1 cells. Outgrowths from 5-d EBs were collected and subcultured 7 days after plating (a). Expansion of MSCs from plating scraped cells with MesenCult; most cells of passage 3–4 exhibited fibroblast-like morphology (b). Bars = 100 μm.

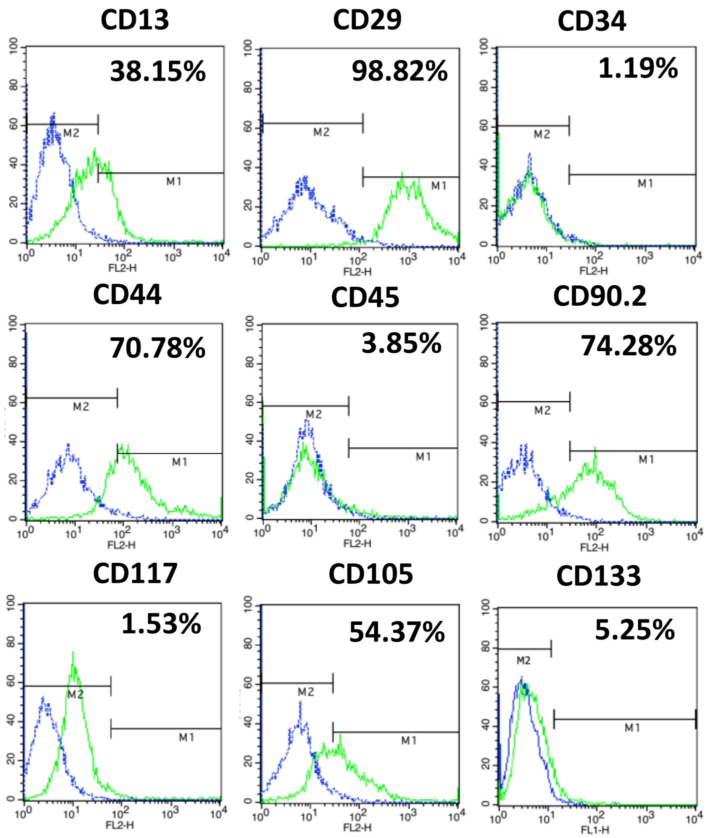

Figure S13. Representative histograms of cell surface marker expression on eMSCs (passage 3) analyzed by flow cytometry. Labeled cells are represented by the green line (M1), and relevant isotype-matched cells are depicted by the blue line (M2).

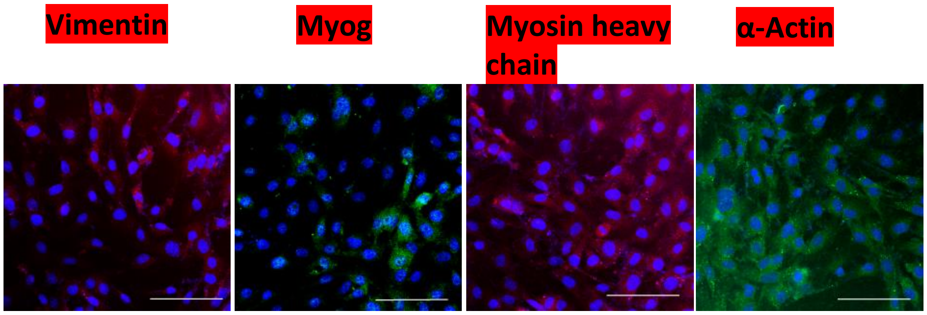

Figure S14. Immunocytochemistry staining for mesodermal markers in eMSCs (passage 3). Antibody staining (green and red), with DAPI nuclear staining (blue). Bars = 100 µm.

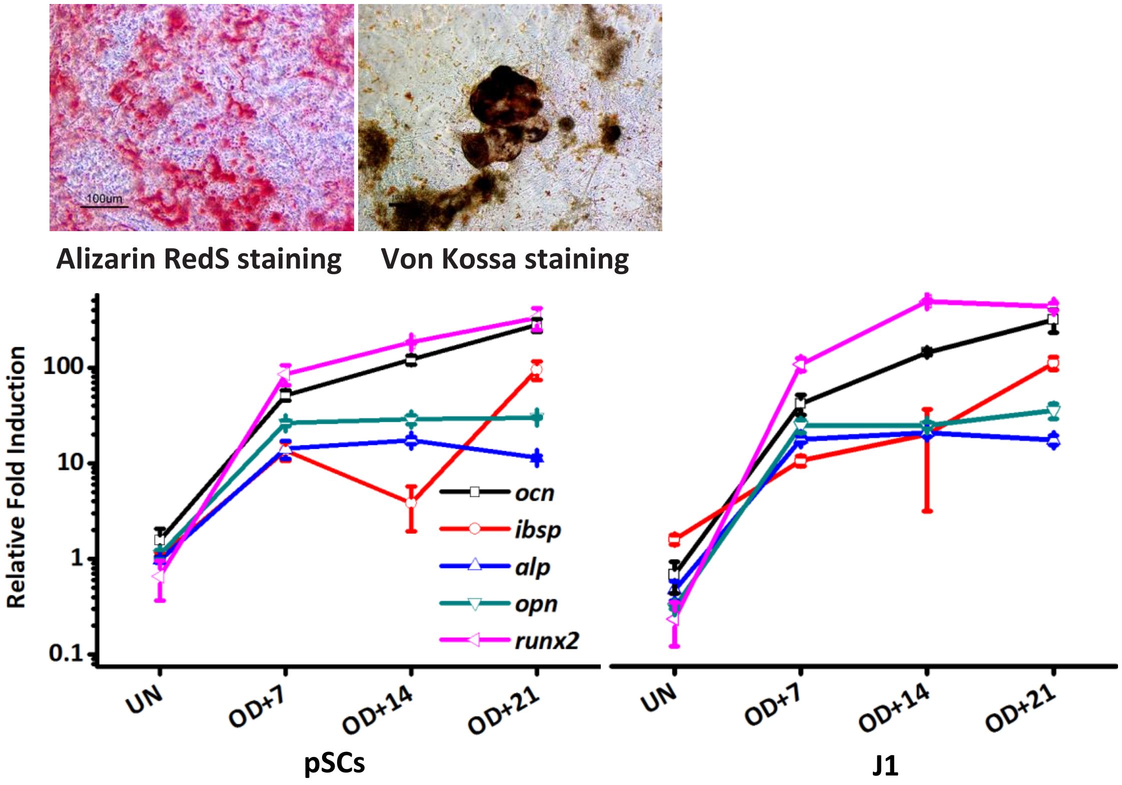

Figure S15. Multidifferentiation potential of putative eMSCs and pMSCs *in vitro*. Alizarin Red S and Von Kossa staining showed the osteogenic differentiation of eMSCs. Bar = 100 µm. Q-PCR was used to determine gene expression profiles related to osteogenic differentiation(OD) compared to uninduced (UN) cells.

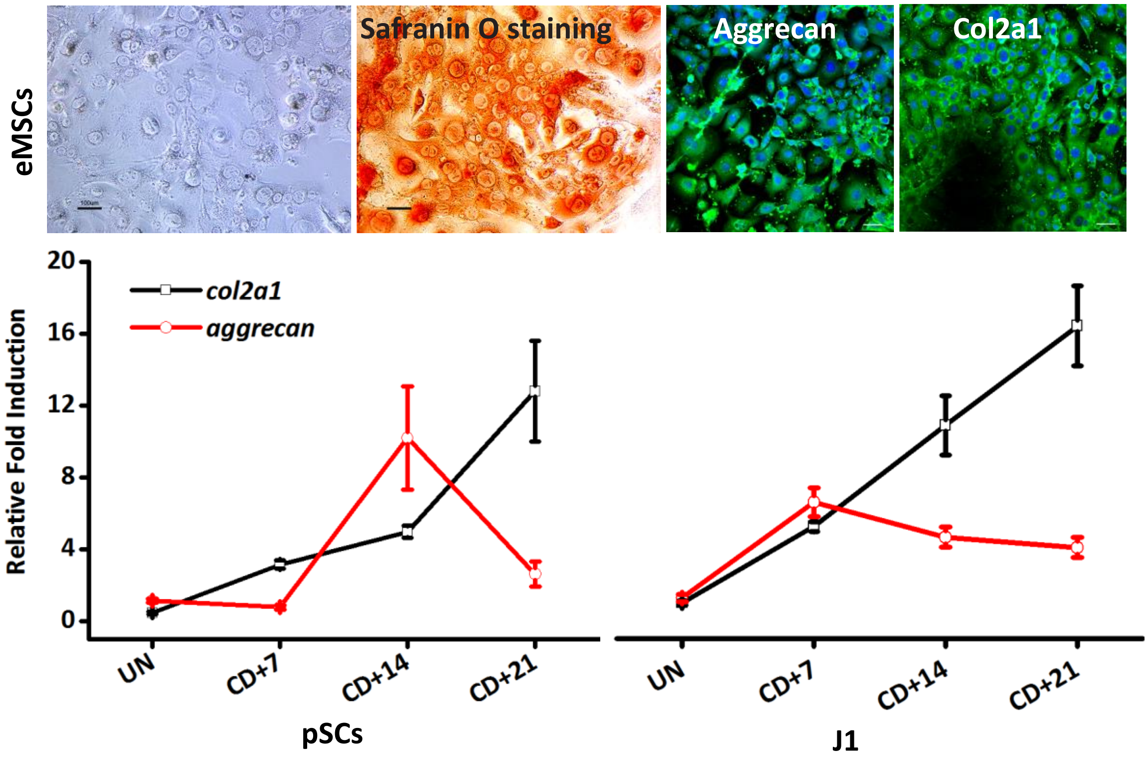

Figure S16. Chondrogenic differentiation of eMSCs. Chondrogenic differentiation was assessed by Safranin O staining and the expression of aggrecan and type II collagen. Bar = 100 µm. Gene expression profiles related to chondrogenic differentiation(CD) in induced and uninduced (UN) cells.

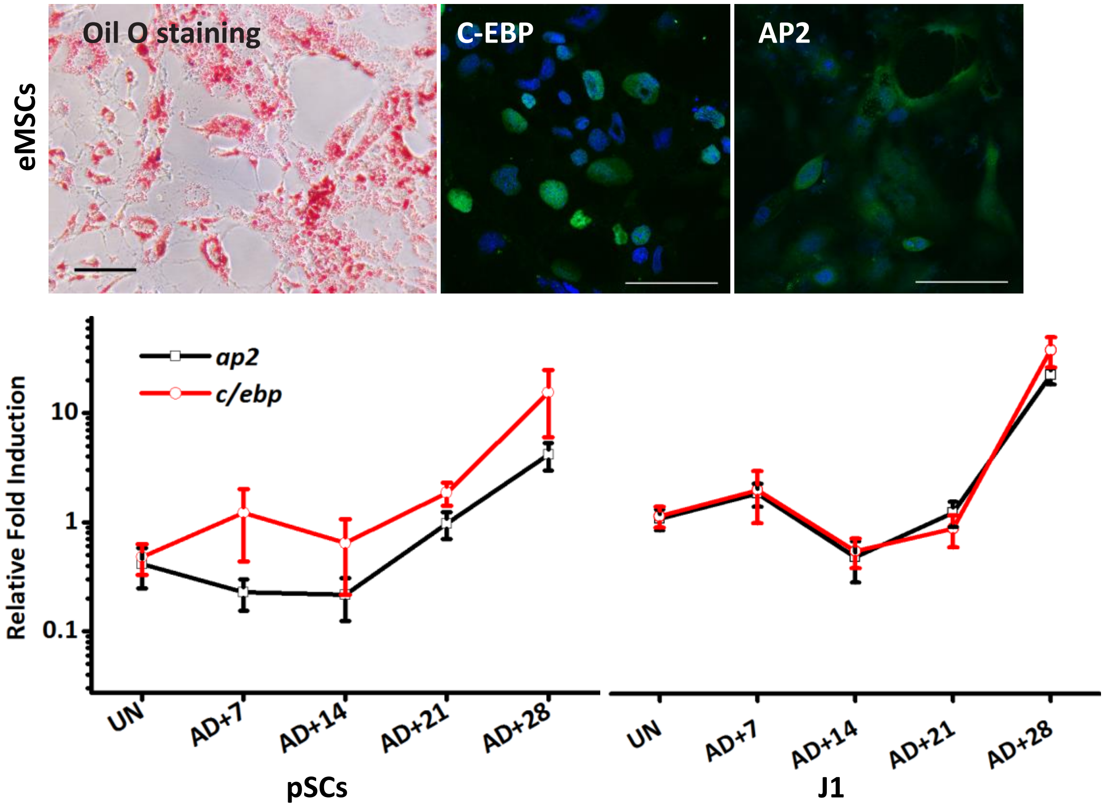

Figure S17. Oil Red O staining showing adipogenic differentiation of eMSCs (top) and pMSCs (bottom). Bar = 100 µm. Gene expression profiles related to adipogenic differentiation(AD) in induced and uninduced (UN) cells.

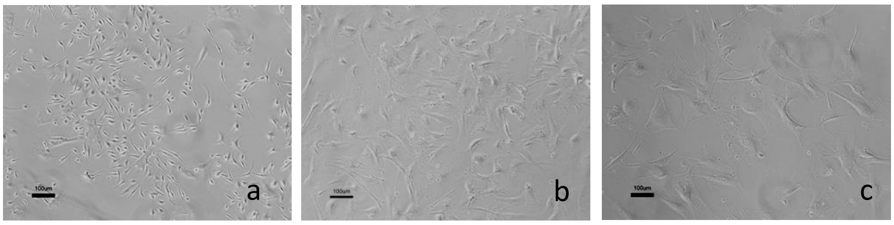

Fig.S18 (A) Morphological observations of primary BMSCs, Fibroblasts and tenocytes under phase-contrast microscopy. Bars = 100 μm.

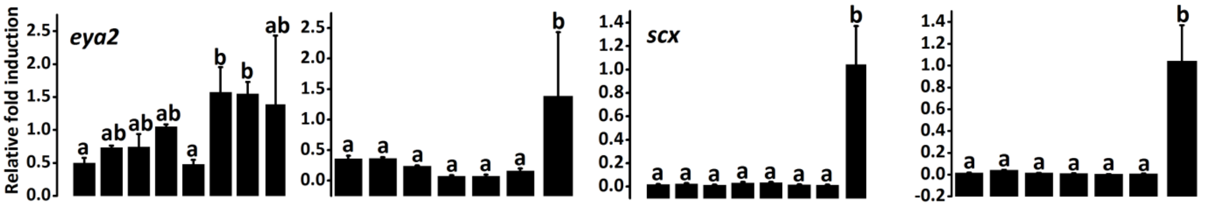

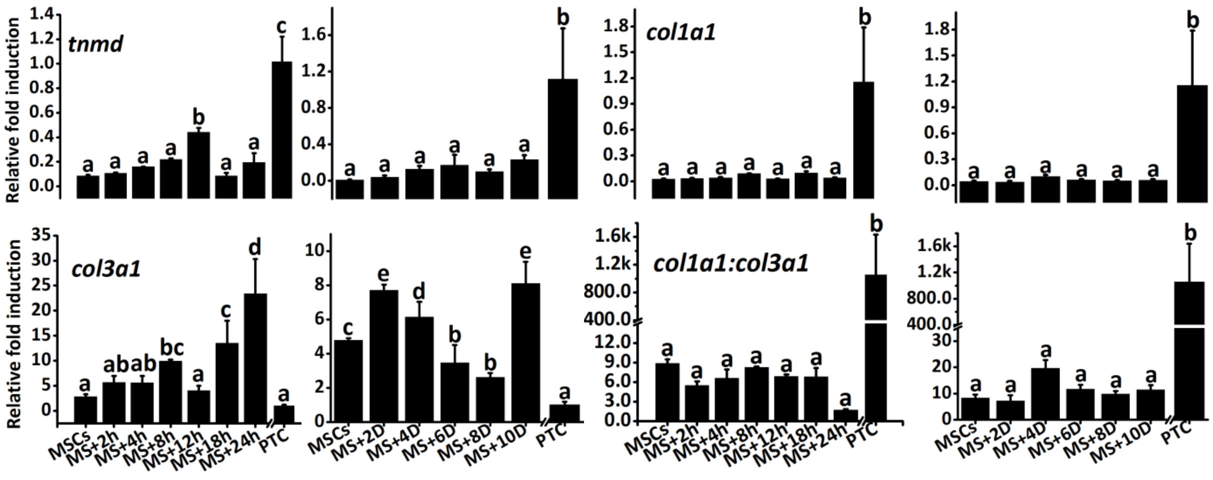

Fig.S19 Q-PCR analysis of the expression of tenocyte-specific markers in MSCs following exposure to 0.1 Hz/10% mechanical stretch for 24 h and 10 days. Graph bars with different letters on top represent statistically significant results ($p<0.05$) based on Newman–Keuls *post hoc* one-way ANOVA analysis, whereas bars with the same letter correspond to results that show no statistically significant differences. In the case where two letters are present on top of the bars in Figure, a, b, c and d represent statistical groupings with differences between each other of $p< 0.05$.

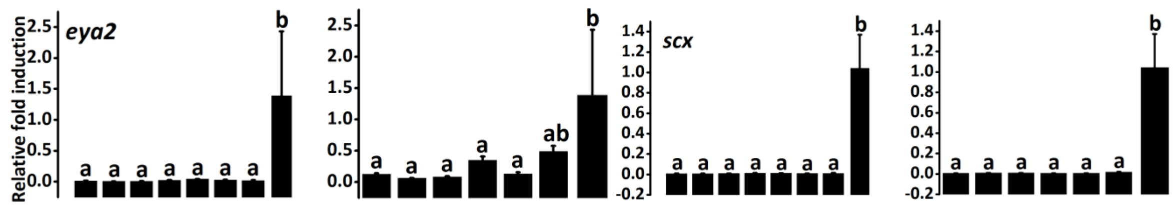

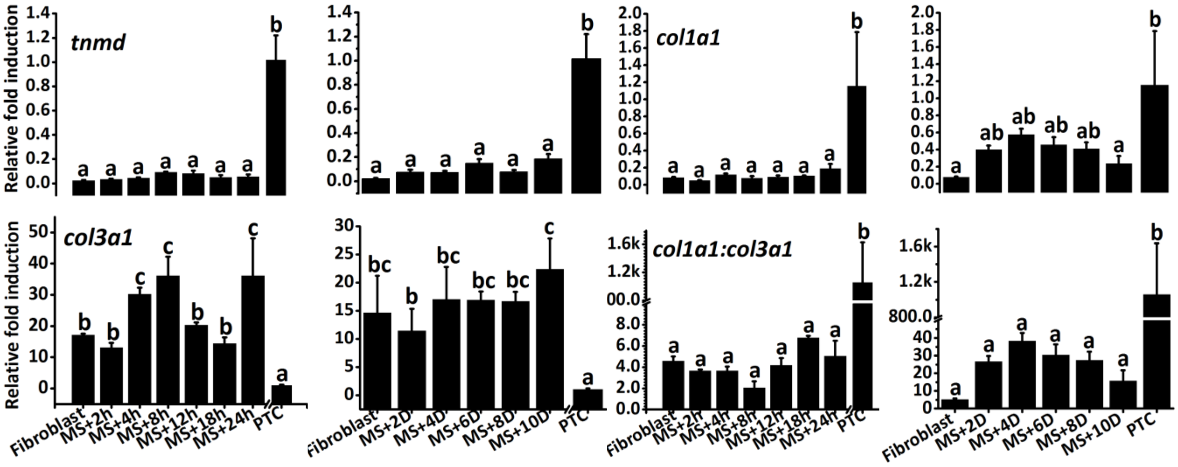

Fig. S20 Q-PCR analysis of the expression of tenocyte-specific markers in Fibroblast following exposure to 0.1 Hz/10% mechanical stretch for 24 h and 10 days. Graph bars with different letters on top represent statistically significant results ($p<0.05$) based on Newman–Keuls *post hoc* one-way ANOVA analysis, whereas bars with the same letter correspond to results that show no statistically significant differences. In the case where two letters are present on top of the bars in Figure, a, b, c and d represent statistical groupings with differences between each other of $p< 0.05$.
